# Vascular Protection of TPE-CA on Hyperhomocysteinemia-induced Vascular Endothelial Dysfunction through AA Metabolism Modulated CYPs Pathway

**DOI:** 10.7150/ijbs.35245

**Published:** 2019-07-25

**Authors:** Hui Li, Zhenli Liu, Linlin Liu, Wen Li, Zhiwen Cao, Zhiqian Song, Qianqian Yang, Aiping Lu, Cheng Lu, Yuanyan Liu

**Affiliations:** 1 School of Chinese Materia Medica, Beijing University of Chinese Medicine, Beijing 100029, China; 2 Institution of Basic Theory, China Academy of Chinese Medical Sciences, Beijing 100700, China; 3 Institute of Basic Research in Clinical Medicine, China Academy of Chinese Medical Sciences, Beijing 100700, China; 4 School of Chinese Medicine, Hong Kong Baptist University, Kowloon, Hongkong, China

**Keywords:** Hyperhomocysteinemia, Vascular endothelial dysfunction, Total phenolic extracts of *Citrus aurantium* L., CYPs signal pathway, Arachidonic acid metabolism

## Abstract

A high concentration of homocysteine (Hcy) in plasma induces vascular endothelial dysfunction, and it may ultimately accelerate the development of cardiovascular diseases (CVDs). Although several B vitamins have been clinically applied for hyperhomocysteinemia (HHcy) treatment, the outcomes are not satisfied due to their limited therapeutic mechanism. Hence, in order to improve the curative effect, development of new effective therapeutic strategies should be put on the agenda. Total phenolic extracts of *Citrus aurantium* L. (TPE-CA) is a naturally obtained phenolic mixture, mainly containing flavones, flavanones and their glycosyl derivatives, flavonols, polymethoxyflavones and coumarins. Previous reports indicated that bioactive phenolic compounds possessed potent vascular protective effects and regarded as a protective agent against CVDs. Intriguingly, the exact mechanism underlying the suppressed effects of TPE-CA on HHcy could assist in revealing their therapy on CVDs. Here, the multi-targeted synergistic mechanism of TPE-CA on HHcy-induced vascular endothelial dysfunction was uncovered in a deduced manner. TPE-CA treatment exhibited an obvious superiority than that of B vitamins treatment. Network pharmacology was employed to identify the interrelationships among compounds, potential targets and putative pathways. Further experimental validation suggested that the treatment of TPE-CA for HHcy could not only effectively reduce the Hcy level in plasma through up-regulating transsulfuration pathway in Hcy metabolism, but also restore the HHcy-induced vascular endothelial dysfunction by activating cytochrome P450 enzymes (CYPs) epoxygenase signal cascades and inhibiting CYPs hydroxylase signal cascades in arachidonic acid (AA) metabolism.

## Introduction

Homocysteine (Hcy) is derived from sulfur-containing and non-proteinogenic amino acid, which occurs as intermediate product during the normal biosynthesis of methionine and cysteine 1. Hyperhomocysteinemia (HHcy) is clinically defined as plasma Hcy higher than 15 µmol/L, which can induce vascular endothelial dysfunction, and is a remarkable and independent risk factor for cardiovascular diseases (CVDs), such as myocardial diseases, coronary disease, and stroke 2, 3. Oxidative stress is a crucial risk leading to vascular endothelial injury attributed to HHcy. Self-oxidation of Hcy produces reactive oxygen species (ROS), which can oxidize low-density lipoprotein (LDL) to oxidized low-density lipoprotein (ox-LDL) and up-regulate the expression of angiotensin converting enzyme (ACE). Moreover, ROS may bind with nitric oxide (NO) to generate ONOO^-^, subsequently induce vascular endothelial dysfunction attributed to HHcy 4.

HHcy has been verified to be involved in vascular endothelial dysfunction via altering the release of vasoactive mediators such as endothelin-1 (ET-1), NO, prostacyclin (PGI_2_), angiotensin Ⅱ (Ang Ⅱ) and thromboxane A_2_ (TXA_2_). Among which, NO and PGI_2_ are two potent endogenous vasodilators synthesized separately by endothelial nitric oxide synthase (eNOS) and prostacyclin synthase (PGIS) in vascular endothelium, and play a critical role as regulator of vascular function 4, 5. TXA_2_ is synthesized by thromboxane A_2_ synthase (TXAS), which acts as a predominant platelet agonist and vasoconstrictor, and promotes platelet aggregation and vasoconstriction 6. ACE plays an essential role in regulation of the vasculature homeostasis. It stimulates the raised constriction in vascular endothelial by catalyzing Ang Ⅰ converted to Ang Ⅱ 7. Moreover, increased Ang Ⅱ leads to excessive production and expression of ET-1, which may accelerate the development of vascular endothelial dysfunction due to its potent vasoconstriction, pro-oxidant and pro-inflammatory properties 8.

Meanwhile, two pathways of remethylation and transsulfuration, promote the metabolism of Hcy and then reduce the Hcy concentration in plasma 9. Accordingly, several catalytic enzymes related to Hcy metabolic pathways, such as methionine synthase (MS), cystathionine-β-synthase (CBS), methylenetetrahydrofolate reductase (MTHFR) and betaine-homocysteine methyltransferase (BHMT) can effectively decrease the plasma Hcy level 10. It is extensively accepted that vitamins B_6_ (pyridoxine), B_9_ (folic acid) and B_12_ (cobalamine) are pivotal co-factors aimed at aforementioned four catalytic enzymes. Due to the deficiency of these co-factors, Hcy remethylation and transsulfuration pathways are hampered, then resulting in HHcy 11. Although many current clinical therapies by supplementing B vitamins may mildly reduce plasma Hcy, the outcomes are not satisfied. B vitamins lower the plasma Hcy level by up-regulating its metabolism, which could only prevent the development of HHcy. Whereas, vascular endothelial injury is triggered in patients with HHcy and subsequently induced vascular endothelial dysfunction cascaded CVDs. Obviously, only focusing on the reduction of Hcy level is insufficient, whilst, exploration of more effective and credible multi-targeted therapeutic strategies for HHcy and its induced vascular endothelial dysfunction is urgently.

Arachidonic acid (AA) derived from membrane phospholipids, which is a free fatty acid that can be metabolized into numerous metabolites. Numerous investigations confirmed that AA metabolism maintained the cardiovascular homeostasis by regulating cardiac and vascular physiology 12. Three pathways such as cytochrome P450 enzymes (CYPs), cyclooxygenase (COX) and lipoxygenase (LOX) are involved in AA metabolism 1. Among them, CYPs pathway contains CYPs epoxygenase signal transduction and CYPs hydroxylase signal transduction, which is considered as the major metabolic pathway of AA metabolism. It is well established that AA metabolic pathways, especially the above mentioned CYPs signal transductions, are involved in vascular endothelial function conducted cardiovascular health and disease 13. Epoxyeicosatrienoic acids (EETs) are generated by CYPs epoxygenase, which possess improvement characters on cardiovascular and vascular endothelial function. However, soluble epoxide hydrolase (sEH) can rapidly hydrolyze EETs to biologically lesser active metabolites, dihydroxyeicosatrienoic acids (DHETs) 13, 14. Therefore, the increase of EETs bioavailability can subsequently protect cardiovascular system and vascular endothelial function via increasing EETs synthesis or reducing EETs hydrolyzation 15. CYPs hydroxylase generates hydroxyeicosatetraenoic acids (HETEs), which are potent vasoconstrictors and the culprit for increasing risk of vascular endothelial dysfunction. Thus, we may reasonably theorize that regulation of CYPs epoxygenase and hydroxylase signal transduction pathways in AA metabolism may exert an effective protection on HHcy-induced vascular endothelial dysfunction, and subsequently hamper the development of CVDs.

The young fruit of *Citrus aurantium* L. named Zhishi has been officially listed in 2015 edition of the Chinese Pharmacopoeia due to its important therapeutic properties for humans. It contains abundant edible bioactive phenolic compounds associated with human health, including flavanones and their glycosyl derivatives, flavones, flavonols, polymethoxyflavones and coumarins, which are commonly authorized nutrient in worldwide. The abovementioned bioactive phenolic mixture named as TPE-CA, was obtained from *Citrus aurantium* L. according to our previous studies 16, 17. Accumulating evidences suggested that bioactive phenolic compounds possessed potent cardiovascular protective effects, and exerted therapeutic effect by regulating blood lipid, blood glucose and vascular endothelial function, and subsequently reduced cardiovascular morbidity and mortality 18, 19. Moreover, Herwandhani Putri found that the flavonoid compounds extracted from *Citrus hystrix* peels exhibited potential cardio-protective effect in clinical use 20. Integrating with our network pharmacology results, indicated that TPE-CA might exhibit multi-targeted synergistic therapeutic effect on HHcy induced vascular endothelial dysfunction.

TPE-CA is a representative multi- compositions and multi-targeted mixture of TCM that performs its synergistic therapeutic efficacy through regulation of the multiple biological processes in body systems. To overcome the bottleneck in TCMs investigation, systematic and comprehensive strategies are required to explore and uncover the multiple therapeutic mechanism of TCMs. In recent years, network pharmacology is used as an effective tool to comprehensively describe complex interactions between complex mixture and biological systems, whilst, it has become a novel and efficient way to uncover the synergistic pharmacological mechanism of TCMs 21, 22. Intriguingly, the concept and core ideas of network pharmacology introduced by Hopkins AL are consistent with the principle of overall regulation of TCMs 23. Therefore, network pharmacology is becoming more conscious for evaluation the multiple therapy on various diseases due to its holistic prediction of the molecular targets and potential pathways of TCMs 24.

In present study, compared with the B vitamins therapy, the protective effects of TPE-CA on HHcy induced vascular endothelial dysfunction were evaluated on the aspects of Hcy levels, enzymes catalyzed Hcy metabolism, vasoactive mediators modulated vascular endothelial function and the histopathology of thoracic aortas. Furthermore, reference to the network pharmacology evaluation, vascular protective mechanism of TPE-CA was elucidated through AA metabolism modulated CYPs epoxygenase and hydroxylase signal cascades. TPE-CA as a naturally occurred mixture, exhibited an obvious superiority than that of B vitamins due to its synergistic therapy.

## Material and Methods

### Reagents and antibodies

Anti-NOX2 (ab31092), Anti-eNOS (ab76198), anti-PGIS (ab23668), anti-Thromboxane synthase (ab187176), anti-BHMT (ab207765), anti-GAPDH (ab8245), anti-Cytochrome P450 2C11 (ab3571) and anti-Cytochrome P450 4A (ab3573) were purchased from Abcam. Anti-sEH (sc-166961) and anti-MTHFR (sc-517229) were obtained from Santa cruz biotechnology, Inc. Anti-CBS (14782S) and anti-MS (68796) were purchased from Cell Signaling Technology. Hcy elisa kit (H245), NO elisa kit (A012-1), ET-1 elisa kit (H093), PGI_2_ elisa kit (H214) and Ang II elisa kit (H185) were purchased from Nanjing Jiancheng Bioengineering Institute. TXA_2_ elisa kit (CEB396Ge) and ACE elisa kit (SEA004Ra) were purchased from Cloud-Clone Corp.. 14,15-EET elisa kit (ab175812) and 20-HETE elisa kit (ab175817) were purchased from Abcam. Secondary antibodies were purchased from ZSGB-Bio. Enhanced BCA protein assay kit (P0010S) was purchased from Beyotime Institute of Biotechnology.

### Preparation of TPE-CA

In present study, the young fruit of *Citrus aurantium* L. was collected from Jiangxi province. And 400 g smashed powder was weighted and dipped in 1 L 50% ethanol for 30min. Then, the dipped powder was boiled 1h per time for three times. The combined aqueous extracts were evaporated and concentrated, afterwards, 132 g of dry extract was obtained through drying in a vacuum oven. These dry extracts were stored at -20 ℃ for further use. The detailed extraction method of TPE-CA was optimized in our previous studies 25.

### Animals and experimental protocol

Current study was performed on male Sprague Dawley (SD) rats (n = 40; 8 weeks old; 180-220 g body weight). All rats were obtained from Beijing Vital River Laboratory Animal Technology Co., Ltd (SCXK (jing) 2016-0011). Rats were maintained in a temperature (22 ℃-25 ℃) and light controlled (06:00 to 18:00) environment and fed with standard laboratory food and water ad libitum. The protocol of the current study was approved by the Research Ethics Committee of Institute of Basic Theory of Chinese Medicine, China Academy of Chinese Medical Sciences (the rodent license NO. SYXK 11-00-0039).

All rats were weighed after acclimatization for 1 week, and then randomly divided into four groups (10 rats per group): normal group, high-methionine diet group (H-Met), TPE-CA group, B vitamins group. Rats in normal group were fed with standard rodent chow in 8-weeks experimental period. To establish HHcy model, a diet added 3 % methionine (w/w) was used to feed rats in H-Met group, TPE-CA group and B vitamins group for 4 weeks 26. Then, TPE-CA group were orally administered with TPE-CA for 4 weeks, the oral daily dose of TPE-CA in rats is 0.6 g / 100 g extract (1.8 g / 100 g crude drug) according to previous experimental work in our laboratory 17. B vitamins group were orally administered with B vitamins (B_6_, 2.1 mg/kg; folic acid, 3.15 mg/kg; B_12_, 0.16 mg/kg) for 4 weeks, these doses were obtained according to the clinical doses for HHcy treatment. The body weights (BW) of rats have been recorded weekly.

### Sample collection

The plasma samples of rats for HHcy evaluation were collected after 4 weeks. 1% pentobarbital sodium was used to intraperitoneally anesthetize rats after 8-weeks experimental period. Rat blood was collected and divided into two parts: one part was collected in common test tube to obtain serum and another was collected in heparinized tube to obtain plasma, and then centrifuged immediately at 10000 rpm for 15 min at 4 ℃. Serum and plasma samples were stored at -80 ℃ for later analysis. The thoracic aortas were removed and dissected into two parts: the part used for Masson's trichrome staining was fixed with 10 % formalin and another part used for later western blot analyzing was stored in -80 ℃ refrigerator. The liver collected from each rat was also stored at -80 ℃ for further analysis.

### Model evaluation

The HHcy model was evaluated by measuring the total plasma Hcy concentrations. Therefore, a commercially available enzyme-linked immunosorbent assay kit for Hcy was applied. The detailed procedures of determination were shown in supplementary material. All procedures were performed in accordance with the manufacturer's specifications.

### Histopathological evaluation

The thoracic aortas removed from each animal group were fixed with 10% neutral buffered formalin, and then embedded in paraffin. After blocks were obtained, samples were sliced into 5μm thick section, then stained with Masson's Trichrome. A light microscope (Olympus, Japan) was applied to histologic observation.

### Determination of target compounds using RRLC-QqQ-MS^n^

Based on our previous study 17, RRLC-QqQ-MS^n^ equipment was applied to determine the compounds contained in TPE-CA. The detailed information was showed in the supporting information.

### Network pharmacology-based analysis

To characterize the interactions among active compounds, targets and pathway, a visualized (compound-target-pathway, C-T-P) network were constructed. Firstly, the candidate targets of active compounds were obtained via virtual fishing in PubChem (https://www.ncbi.nlm.nih.gov/), STITCH (http://stitch.embl.de/, ver, 5.0) and Traditional Chinese Medicine Systems Pharmacology Database and Analysis Platform (TCMSP, http://lsp.nwu.edu.cn/tcmsp.php, ver, 2.3). Compounds and their related candidate target proteins linked with an edge. Afterwards, the mean degree of targets was employed to screen the candidate targets, and then the screened targets were imported to STRING database (http://string-db.org/, ver, 11.0) and the Database for Annotation, Visualization and Integrated Discovery (DAVID, https://david.ncifcrf.gov/home.jsp, ver, 6.8) for protein-protein interactions analysis and pathway prediction, respectively. Then, the pathway information of targets was extracted from Kyoto Encyclopedia of Genes and Genomes (KEGG, http://www.kegg.jp). Finally, Cytoscape software (http://www.cytoscape.org/, ver, 3.7.0) was applied to construct the bipartite visualized C-T-P network.

### Determination of nitric oxide (NO) in rat plasma

NO decomposes rapidly to form stable metabolite nitrite (NO_2_^-^) and nitrate (NO_3_^-^) in vivo. To detect the precise concentration of NO in plasma, both NO_2_^-^ and NO_3_^-^ concentrations should be precisely measured. The detailed procedure according to the manufacturer's specifications was supplied in the supporting information.

### Enzyme-linked immune sorbent assay

Enzyme-linked immune sorbent assay (ELISA) kits were applied to measure the concentration of ET-1, TXA_2_, PGI_2_, Ang Ⅱ, 14, 15-EET, 20-HETE and ACE in rat blood. For instance, the rat plasma samples were used for determination of ET-1, TXA_2_, PGI_2_, Ang Ⅱ, 14, 15-EET and 20-HETE, and the rat serum samples were used for determination of ACE expression level. All assay kits were applied competition method. The detailed procedures of determination were shown in supplementary material. All procedures performed base on the manufacturer's specifications.

### Western blot analysis

Total proteins were extracted from thoracic aortic endothelial tissue and liver via homogenization at 4 ℃ in RIPA buffer supplemented with protease and phosphatase inhibitors. Obtained homogenates were centrifuged at 13000 rpm at 4℃ for 20 min, and then collected the supernatant. The protein concentration of the supernatant was measured using the enhanced BCA protein assay kits, and then the protein was stored at -80 ℃ for further analysis.

Proteins were separated on a sodium dodecyl sulfate-polyacrylamide gel electrophoresis (SDS-PAGE); the sample volume was 50 μg for each well. After that, the proteins were electro-transferred to polyvinylidene difluoride (PVDF) membrane, then the membrane was blocked for 2 h using 5% non-fat milk at room temperature. Afterwards, the membranes were incubated with specified antibodies overnight at 4 ℃: GAPDH (1:5000 dilution), NOX2 (1:1000 dilution), MTHFR (1:500 dilution), MS (1:1000 dilution), CBS (1:1000 dilution), BHMT (1:1000 dilution), CYP2C11 (1:1500 dilution), CYP4A (1:1000 dilution), sEH (1:500 dilution), eNOS (1:1000 dilution), PGIS (1:250 dilution), TXAS (1:1000 dilution), followed by incubation with horseradish peroxidase-conjugated secondary antibodies (1:5000) at room temperature for 2 h. After that, the protein bands were visualized by enhanced chemiluminescence detection reagents. Band intensity was calculated as follows: sum of all pixel values - background pixel value = band intensity. Protein expression levels were normalized to GAPDH.

### Statistical analysis

The results were calculated as means ± standard deviation (SD). Graphpad software was employed for one-way analysis of variance (ANOVA) and comparisons between groups. Tukey's multiple comparison tests were utilized when data were normally distributed, non-parametric tests were utilized when data were not normally. p < 0.05 were considered statistically significant. (*p < 0.05, **p < 0.01, ***p < 0.001)

## Results

### Effects of TPE-CA on rat's growth

**Figure 1:**
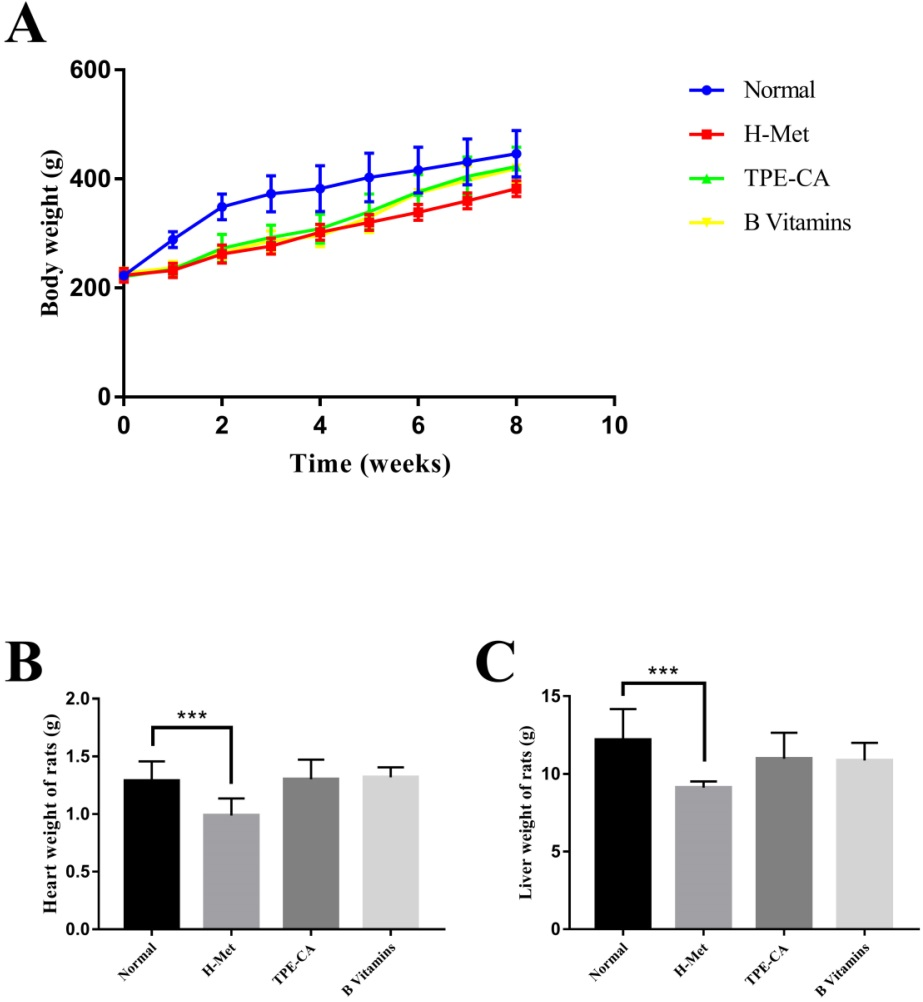
Effects of TPE-CA and B vitamins on the body, heart and liver weight of rats.

The BW of rats was recorded weekly, and the organ weight of heart and liver was recorded after all rats were sacrificed. As shown in Fig.1A. The H-Met group, TPE-CA group and B vitamins group had lower weight gain during the model induction period. However, it was ameliorated when treatment with TPE-CA and B vitamins, respectively. Compared with the normal group, both TPE-CA group and B vitamins group exhibited slight significant difference in heart and liver weight but it was significantly reduced in H-Met group (Fig. 1B and C).

### Concentration of Hcy in rat plasma

**Figure 2:**
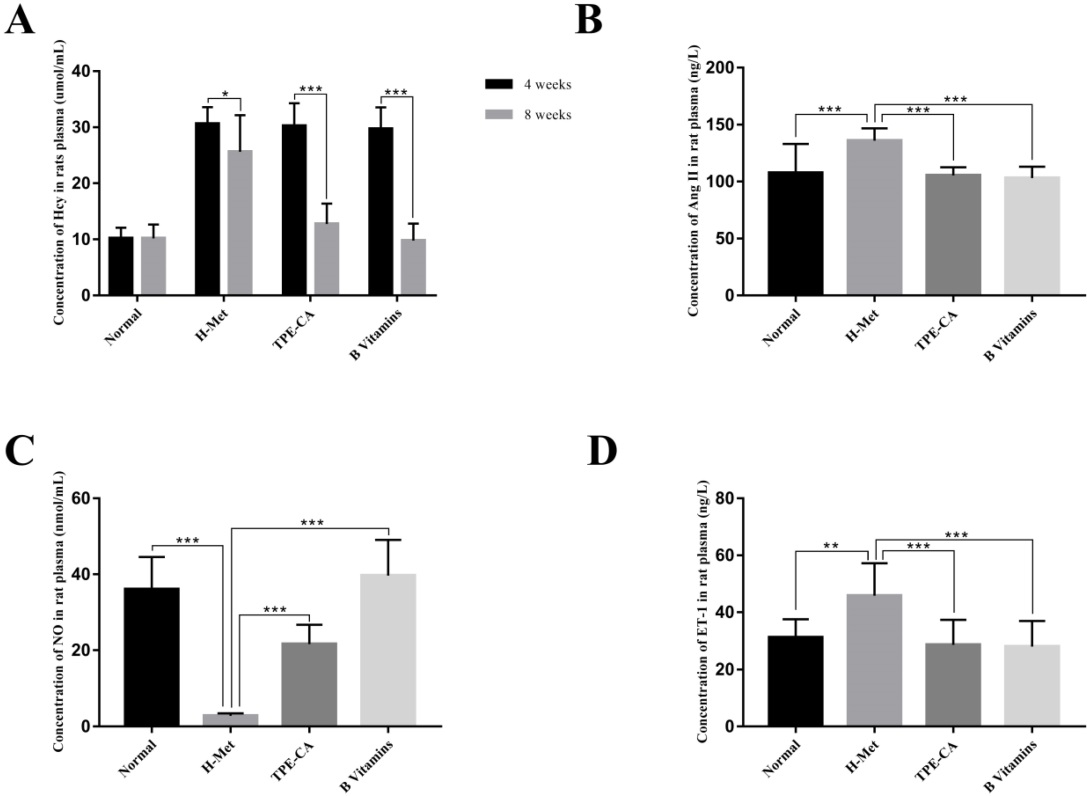
Effects of TPE-CA and B vitamins on Hcy level and the release of vasoactive mediators in rat's plasma. (A) The concentrations of Hcy. (B) The concentrations of Ang Ⅱ. (C) The concentrations of NO. (D) The concentrations of ET-1.

HHcy is clinically defined as plasma Hcy higher than 15 µmol/L. Substantial evidences indicate that HHcy-induced vascular endothelial dysfunction is ascribed to an increased Hcy concentration. Therefore, the plasma Hcy concentration, as a pivotal index for model evaluating, was measured by using a commercially available Elisa kit. As shown in Fig. 2A. After 4 weeks, the levels of Hcy in plasma was significantly increased in H-Met group, TPE-CA group and B vitamins group that higher than 15 μmol/L. It indicated that the HHcy model has been induced successfully in these three groups. After 4 weeks of treatment, the Hcy concentrations in both TPE-CA group and B vitamins group were significantly decreased and lower than 15 μmol/L.

### Effects of TPE-CA on vasoactive mediators

ET-1, Ang Ⅱ and NO play different roles in vascular functions, among which ET-1 and Ang Ⅱ are potent vasoconstrictor and accelerate the development of vascular endothelial dysfunction. While, NO is an endothelium derived relaxing factor and plays a key role in regulating vascular tone. Thereby, the release of these three vasoactive mediators was examined in this study. As shown in Fig. 2, compared with normal group, ET-1 and Ang Ⅱ concentrations in rat plasma were significantly increased in H-Met group (Fig. 2B and D). Whereas, NO concentration was significantly reduced in the H-Met group (Fig. 2C). Comparatively, ET-1 and Ang Ⅱ levels in plasma were significantly lower in TPE-CA group and B vitamins group than these in H-Met group. Plasma level of NO was significantly higher in TPE-CA group and B vitamins group than that of the H-Met group. Above results demonstrate that TPE-CA can ameliorate the vascular endothelial dysfunction by regulating the release of vasoactive mediators.

### Histopathological evaluation

**Figure 3:**
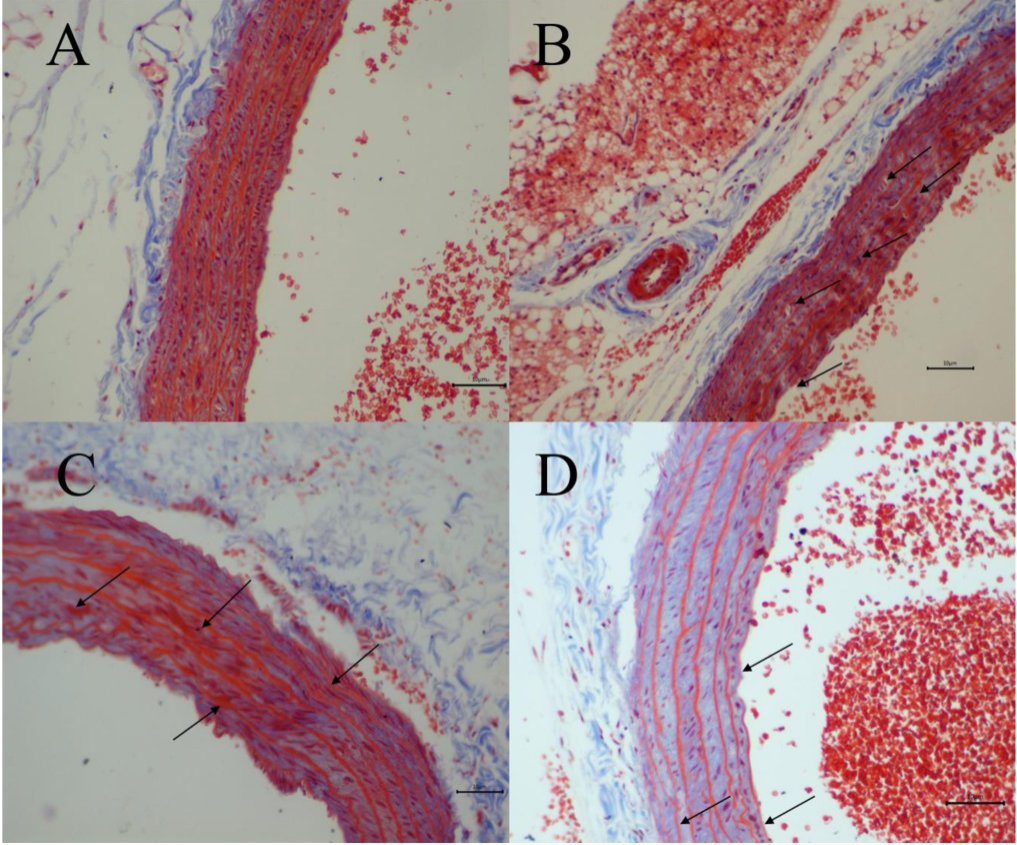
Vascular histopathology analysis (Magnification: 200×). (A) Normal group. (B) H-Met group. (C) TPE-CA group. (D) B vitamins group. The marked histopathological characteristic in each group was indicated by arrowhead. Scale bar = 10μm.

The degree of vascular injury was observed by Masson's Trichrome staining under light microscope. Fig. 3 depicted representative histologic sections of Masson's Trichrome. A vascular wall with normal histoarchitecture, including adventitia, middle, and inner layers, was observed in the normal group (Fig. 3A). However, in H-Met group, the histoarchitecture was destroyed, and the myofiber contained in vascular wall was markedly degraded and broken, and collagen was dissolved and lost (Fig. 3B). In TPE-CA group, it exhibited an obvious repair state in myofiber rupture, collagen proportion and the vascular wall architecture (Fig. 3C). Similarly, these vascular injuries have been attenuated in B vitamins group as shown in Fig. 3D. However, the degree of recovery in B vitamins group was lower than TPE-CA group. Above results showed that rats received TPE-CA or B vitamins treatment could improve and restore the damaged vascular wall histoarchitecture.

### Effects of TPE-CA on remethylation pathway and transsulfuration pathway

**Figure 4:**
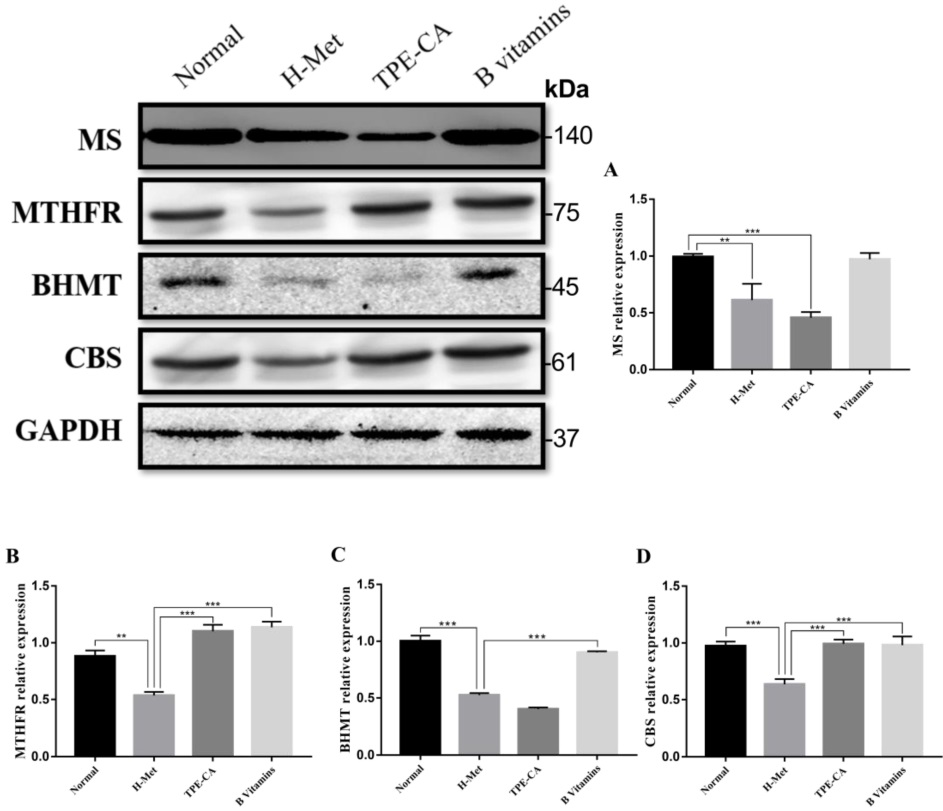
Effects of TPE-CA and B vitamins on the pivotal proteins of Hcy metabolism. (A) The relative expression of MS. (B) The relative expression of MTHFR. (C) The relative expression of BHMT. (D) The relative expression of CBS. Protein expression levels were normalized to GAPDH.

The abovementioned results indicated that TPE-CA treatment could significantly attenuate HHcy. To understand the mechanism of TPE-CA treatment on HHcy model in remethylation and transsulfuration pathway, the pivotal protein levels including MS, MTHFR, BHMT and CBS were investigated using western blotting. As shown in Fig. 4, compared with the normal group, the protein expression levels of MS, MTHFR, BHMT and CBS were significantly reduced in the H-Met group, whereas, they were significantly increased in B vitamins group compared with the H-Met group. The levels of MTHFR and CBS were significantly increased in TPE-CA group compared with the H-Met group, but there were no significant differences in the protein expression levels of MS and BHMT between TPE-CA group and H-Met group. Above results showed that TPE-CA might ameliorate the Hcy level via activating the transsulfuration pathway but rarely in remethylation pathway.

### Determination of TPE-CA by RRLC-QqQ-MS^n^ analysis

RRLC-QqQ-MS^n^ was performed for quantitative determination of chemical constituents in TPE-CA. The corresponding method validation has been summarized in our previous studies 25. MRM chromatograms of 32 compounds were shown in Fig. S1. A total of 32 compounds were detected, including kaempferitrin, rutin, eriocitrin, diosmetin-7-O-glucoside, narirutin, apigenin-7-O-glucoside, rhoifolin, naringin, diosmin, hesperidin, neohesperidin, xanthotoxol, scoparone, poncirin, eriodictyol, luteolin, apigenin, naringenin, diosmetin, hesperetin, sinensetin, isopimpinellin, limonin, bergapten, isosakuranetin, nomilin, acacetin, nobiletin, tangeretin, 5-demethylnobiletin, auraptene, imperatorin. The information for MRM parameters and the contents of those compounds in TPE-CA have been shown in Table S1 and S2.

### Network pharmacology-based analysis

**Figure 5:**
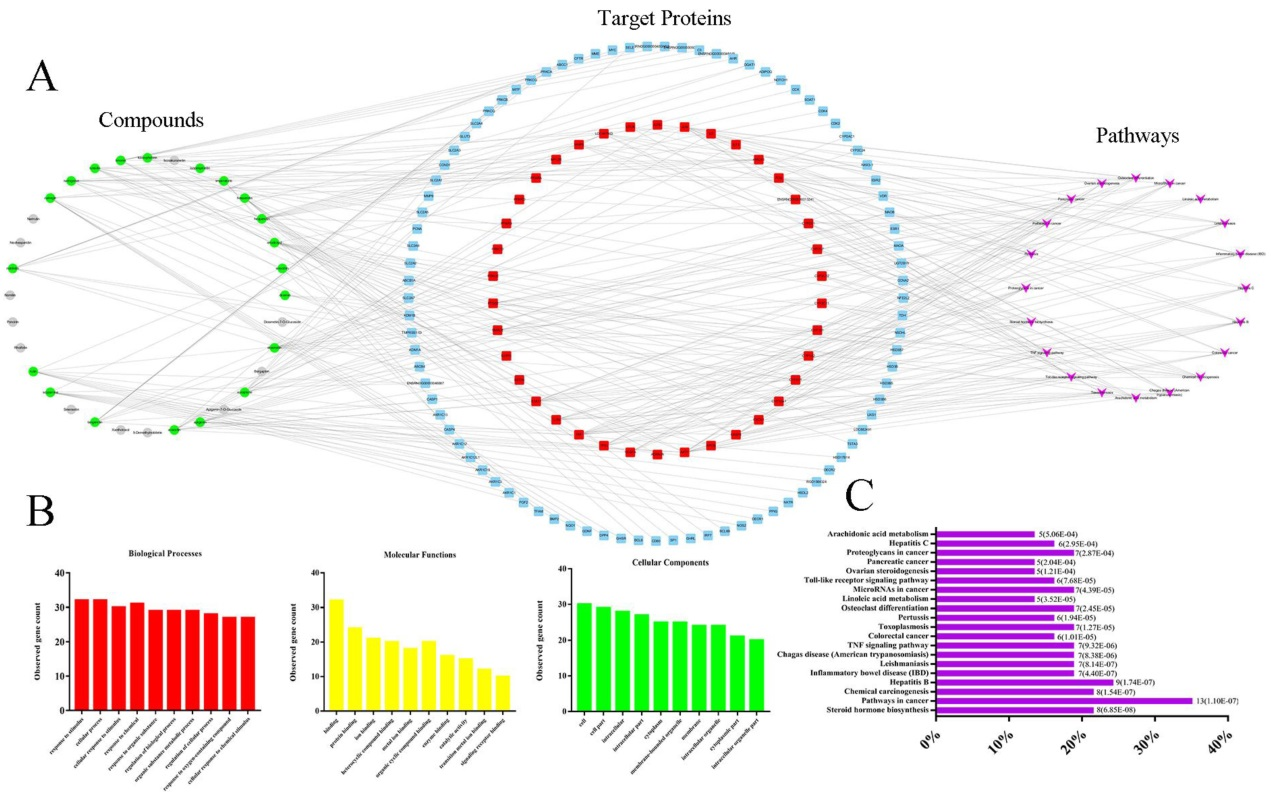
Network pharmacology-based analysis of 32 active compounds contained in TPE-CA. (A) The Component-Target-Pathway network. The green circles, gray circles, blue round rectangle, red round rectangle and purple quadrangle corresponded to compounds with potential targets, compounds without potential targets, candidate targets, screened candidate targets and putative pathways, respectively. (B) The enrichment analysis of 38 target proteins by STRING database. (C) The enrichment analysis of target proteins related diseases by DIVAD database.

Current C-T-P network was established by active compounds, potential targets and corresponding pathways. This network contained 179 nodes (32 active compound nodes, 127 candidate target nodes and 20 putative pathways) and 303 edges (Fig. 5A), in which green circles, gray circles, blue round rectangle, red round rectangle and purple quadrangle corresponded to compounds with potential targets, compounds without potential targets, candidate targets, screened candidate targets and putative pathways, respectively. To select the targets with higher impact in C-T-P network, the mean degree of all targets was calculated according to the Table S3 (in current experiment, mean degree = 4). Therefore, the threshold to screen targets was set to 5, and 38 targets were selected and placed in the middle of network. Pathways information obtained from DAVID and KEGG were listed in Table S4, and the top 20 pathways were selected for further analysis. Reference to previous literature study, AA metabolism maintained cardiovascular homeostasis through modulating cardiac and vascular physiology. Thus, AA metabolism was picked for further experimental validation. Indeed, accumulating evidence suggests that CYP2C mediated CYPs epoxygenase signal transduction in AA metabolism exerted diverse protective effects on vascular endothelial function 14, 27, 28. In addition, in order to completely uncover the roles of CYPs pathway of AA metabolism in HHcy rat with TPE-CA treatment, the CYP4A mediated CYPs hydroxylase signal transduction also be investigated in present study.

The biological processes, molecular functions, and cellular components of target proteins were analyzed through STRING database. The interactions of target proteins analyzed by STRING database were summarized in Table S5. The biological processes, cellular components, and molecular functions with P value < 0.05 were considered to be meaningful, as shown in Fig. 5B. Biological processes of the target proteins were mainly related to response to stimulus and cellular process. Banding activity was mainly contained in molecular functions of target proteins, such as protein binding and ion binding. In addition, based on the classification analysis of cellular components, the target proteins located in the cell, intracellular and cytoplasm. Then, we analyzed the categories of related diseases, the results are showed in Fig. 5C. We found that cancer, metabolic diseases, hepatitis, inflammatory diseases and infectious diseases were the main disease categories. In current study, AA metabolism as a metabolic disease related metabolic pathway was picked for further investigation.

### Reversion of TPE-CA on imbalanced PGI_2_ and TXA_2_ and their related synthase of PGIS and TXAS

**Figure 6:**
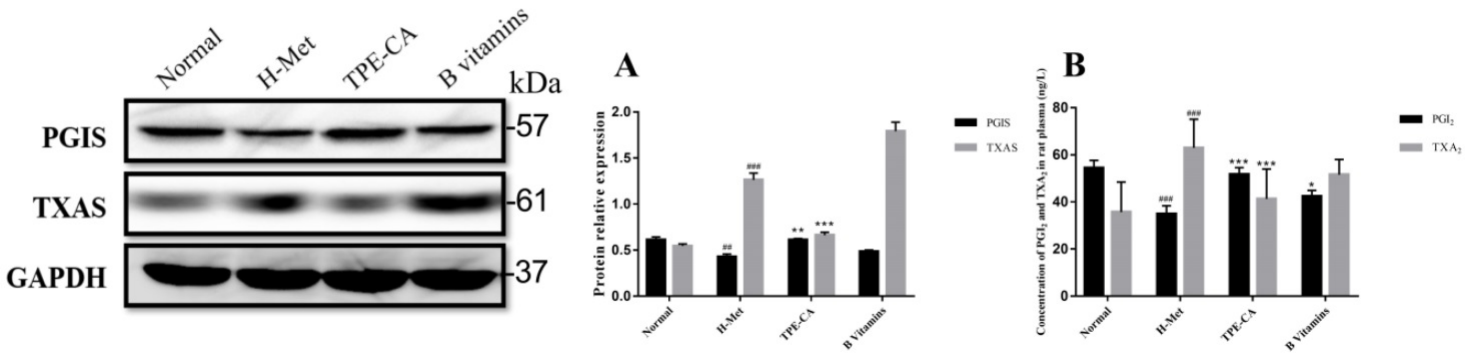
Effects of TPE-CA on the balance of PGI_2_ / TXA_2_ and PGIS / TXAS. (A) The relative expression of PGIS and TXAS. (B) The concentration of PGI_2_ and TXA_2_. **#**p<0.05 vs Normal group, **##**p<0.01 vs Normal group, **###**p<0.001 vs Normal group. *p<0.05 vs H-Met group, **p<0.01 vs H-Met group, ***p<0.001 vs H-Met group.

PGI_2_ and TXA_2_ exert opposite effects in the homeostasis of vasculature and platelet aggregation, and the balance in PGI_2_ and TXA_2_ play a crucial role in correct vascular endothelial function. In present study, the levels of PGI_2_ and TXA_2_ in rat plasma were detected. In addition, the expression of their related synthase as PGIS and TXAS were detected using western blotting. As depicted in Fig. 6A and B, the marked imbalance of PGI_2_ and TXA_2_ were existed in H-Met group ascribed to decreased PGI_2_ and increased TXA_2_. Meanwhile, the western blotting bands showed that the expression of PGIS was reduced but the TXAS was elevated in H-Met group. In B vitamins group, the imbalance in both PGI_2_ / TXA_2_ and PGIS / TXAS were slightly eliminated. However, these imbalances were significantly reversed in TPE-CA group. It prompted that TPE-CA treatment possessed a superiority than that of B vitamins.

### Effects of TPE-CA on self-oxidation of Hcy

**Figure 7:**
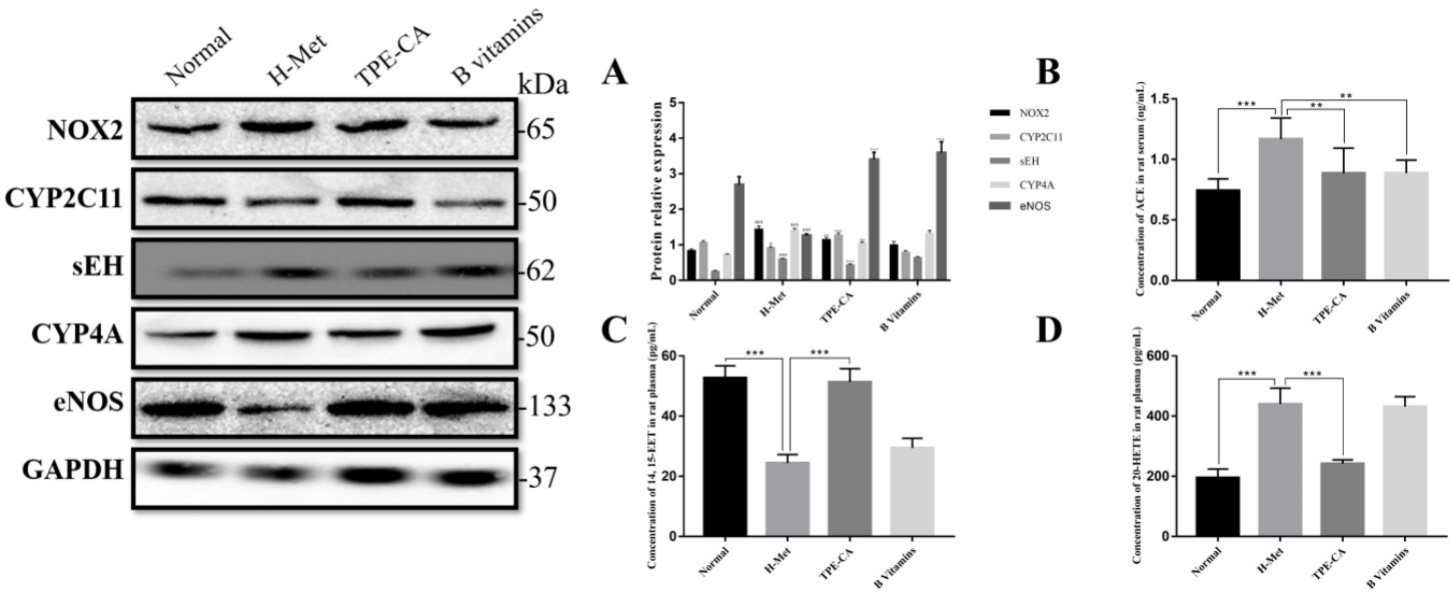
Effects of TPE-CA on the CYPs epoxygenase and CYP hydroxylase signal transduction of AA metabolism. (A) The relative expression of NOX2, CYP2C11, sEH, CYP4A and eNOS. (B) The concentration of ACE. (C) The concentration of 14, 15-EET. (D) The concentration of 20-HETE. **#**p<0.05 vs Normal group, **##**p<0.01 vs Normal group, **###**p<0.001 vs Normal group. *p<0.05 vs H-Met group, **p<0.01 vs H-Met group, ***p<0.001 vs H-Met group.

Previous report indicated that NADPH oxidase 2 (NOX2) was the major source of ROS in endothelial cell. Thus, the expression level of NOX2 was detected by western blotting in present study. As shown in Fig. 7A, the relative expression of NOX2 in H-Met group was markedly increased. Whereas, the expression of NOX2 was significantly inhibited in TPE-CA and B vitamins group. Both the TPE-CA and B vitamins treatment could significantly diminish the oxidative stress in vascular endothelial.

### Activation of TPE-CA on CYPs epoxygenase pathway signal transduction

Based on the network pharmacology analysis and previous literature investigation, we focused on CYP2C mediated CYPs epoxygenase pathway signal transduction of AA metabolism to discover the potential therapeutic mechanism of TPE-CA. In Fig. 7C, 14,15-EET content in rat plasma was significantly decreased in H-Met group and B vitamins groups compared with normal group. Whereas, it was significantly increased in TPE-CA group. Moreover, the western blot analysis results of H-Met group showed that the protein expression of CYP2C11 were inhibited but sEH were activated. This status was reversed in TPE-CA group, as shown in Fig. 7A, the protein expression of CYP2C11 have a significant activation, while the protein expression level of sEH was significantly decreased. The results illustrated that TPE-CA could effectively activate CYPs epoxygenase pathway signal transduction and inhibit the process of 14,15-EET converted to 14,15-DHET. However, above actions were not markedly observed in B vitamins group.

### Inhibition of TPE-CA on CYPs hydroxylase pathway signal transduction

To investigate the mechanism of the CYPs hydroxylase pathway signal transduction in HHcy model with TPE-CA treatment, the protein expression level of CYP4A were determined. In addition, the level of 20-HETE, which formed from arachidonic acid by CYPs hydroxylase pathway, was determined in plasma. Previous studies have suggested that 20-HETE led to the activation of ACE and the inactivation of eNOS 29, which subsequently triggers vascular endothelial dysfunction. Thus, we evaluated these enzymes using western blotting. As shown in Fig. 7A and Fig. 7D, both the protein expression level of CYP4A and the 20-HETE concentration were significantly higher in H-Met group than that of normal group and TPE-CA group. While B vitamins group was closed to H-Met group. In Fig. 7B, the protein expression levels of ACE were increased, yet the eNOS expression level was decreased in H-Met group. Whereas, these findings were reversed by TPE-CA and B vitamins treatment. Above results confirmed that TPE-CA exerted vascular protection by inhibiting CYPs hydroxylase pathway signal transduction and reducing the production of 20-HETE.

## Discussion

High concentration of Hcy triggers vascular endothelial dysfunction in patients, and ultimately lead to CVDs, which effects about 30 % of the Western population in recent decades. It is indeed commonly accepted that two metabolic pathways including remethylation pathway and transsulfuration pathway can reduce the Hcy level by converting Hcy to methionine and cysteine, respectively. Supplementation of vitamin B_6_, vitamin B_9_ and vitamin B_12_ as a common therapeutic method, is applied frequently in clinical, to promote the metabolism of Hcy and then reduce the Hcy level. However, B vitamins applied to reduce plasma Hcy level in many clinical trials were not satisfied 30. Because HHcy-induced vascular endothelial dysfunction might still accelerate the development of CVDs. Thus, it is urgently to develop some emerging therapeutic strategies for HHcy-induced vascular endothelial dysfunction. In present study, we used a diet-induced HHcy model to imitate the clinical situation of HHcy. Then, the basic anthropometric characteristics of rats were initially examined 31, 32. It initially indicated that the treatment of TPE-CA and B vitamins for 4 weeks might mitigate the high-methionine diet induced body, heart and liver lose.

Vascular endothelium plays vital roles in the management of microvascular permeability, coagulation, inflammation and vascular tone. These functions are exerted via releasing variety of vasoactive mediators 3. Many reports showed that NO and PGI_2_ were two potent endogenous vasodilators, who exerted diverse protection on blood vessel such as anti-platelet aggregation, anti-proliferation, anti-atherogenic and so on 3, 5. Whereas, vasoactive mediators such as ET-1, Ang Ⅱ and TXA_2_ were play a negative role in vascular endothelial functions. They hurt blood vessel by promoting platelet aggregation, provoking vasoconstriction, increasing oxidative stress as well as promoting inflammation 6-8. In current experiment, to explore the effects of TPE-CA on HHcy-induced vascular endothelial dysfunction, abovementioned five vasoactive mediators were measured. Our data indicated that TPE-CA treatment could effectively lighten the HHcy-induced vascular endothelial dysfunction. TPE-CA exerted vascular protection via reducing the plasma concentration of ET-1, Ang Ⅱ and TXA_2_, while, increasing the concentration of NO and PGI_2_ in plasma. Thereby, the imbalance among these vasoactive mediators were removed by TPE-CA treatment.

The B vitamins could reduce the in vivo Hcy level through MS modulated remethylation pathway and CBS modulated transsulfuration pathway of Hcy metabolism. Hcy remethylation required B vitamins and 5,10-MTHF, which was generated by MTHFR. Hcy transsulfuration also required B vitamins. In liver, some Hcy was remethylated to methionine through an alternative pathway catalyzed by BHMT. Our finds showed that all metabolic enzymes had been well activated and the expression level significantly increased with B vitamins treatment. However, TPE-CA could only activate the expression of MTHFR and CBS but rarely in the MS and BHMT. Although TPE-CA had activated MTHFR, MS-mediated remethylation pathway showed no significant activation ascribed to inactivated MS. The expression of CBS was increased by TPE-CA treatment followed by increased transsulfuration of Hcy to cystathionine, and then the Hcy level was reduced in vivo. It demonstrated that the ameliorative effects of TPE-CA on HHcy were mainly manipulated by activating CBS mediated transsulfuration pathway.

TPE-CA could not only improve the body, heart and liver weight of rats with HHcy, but also correctly regulate the release of several vasoactive mediators. Moreover, in histopathological examination, TPE-CA showed marked amelioration on the HHcy induced histoarchitecture injury in vascular wall. To investigate the potential vascular protective mechanisms of TPE-CA, a network pharmacology-based analysis, which was regarded as a potent tool to comprehensively describe complex interactions between complex mixture and biological systems 33, was employed to unravel the potential vascular protective mechanism of TPE-CA. In current study, 32 characteristic compounds from TPE-CA were used to find putative binding proteins by virtual fishing. AA metabolism as a metabolic disease related metabolic pathway, maintained the cardiovascular homeostasis by regulating cardiac and vascular physiology, was picked for further investigation.

CYPs epoxygenases metabolized AA into four regioisomers of EETs, including 5,6-EET, 8,9-EET, 11,12-EET and 14,15-EET, among which 14,15-EET were quantified as the primary metabolic product synthesized by CYP2C 34. EETs has several beneficial effects on vascular tone and cardiovascular homeostasis, including dilating coronary arteries, suppressing adhesion molecules and hyperpolarizing vascular smooth muscle cells. Whereas, sEH enzyme, as a crucial determinant of EETs level, catalyzed the conversion of EETs to DHETs, and then abolished the biological activity of EETs. Thus, sEH enzyme had gained considerable attention as a new therapeutic target for vascular endothelial dysfunction in resent year 35. 20-HETE was a CYPs hydroxylase-derived metabolite of AA, involved with the renin-angiotensin system (RAS) to promote hypertension, vasoconstriction, and vascular dysfunction 36. In addition, 20-HETE was able to increase the expression of ACE, subsequently raised the Ang Ⅱ concentration in vivo. What is more, 20-HETE would trigger the uncoupling of eNOS, followed by reduction of NO bioavailability, and then induced vascular endothelial dysfunction 29. Also, 20-HETE activated NOX2 and then promoted the production of ROS, then oxidative stress was induced in vascular endothelial cell. In this light, we could infer that CYPs hydroxylase signal cascade may be an underlying mechanism of HHcy to induce vascular endothelial dysfunction. Thus, the regulation between CYPs epoxygenase signal cascade and CYPs hydroxylase signal cascade played a critical role in vascular endothelial dysfunction.

According to the results of present experiment, we found that TPE-CA markedly ameliorated HHcy-induced vascular endothelial dysfunction. On one hand, TPE-CA significantly up-regulated the expression level of CYP2C11 followed by activation of the CYPs epoxygenase pathway signal cascade, and then enhanced EETs level in vivo. In addition, sEH as a considerable therapeutic target was inhibited by TPE-CA in our present study. It is noteworthy that TPE-CA could markedly increase EETs contents by not only improving the synthesis of EETs, but also inhibiting the metabolism of EETs. Therefore, TPE-CA might be a considerable therapeutic agent for HHcy-induced vascular endothelial dysfunction. On the other hand, CYP4A, which possessed the greatest catalytic activity in CYPs hydroxylase signal cascade 29, was repressed by TPE-CA. In doing so, CYPs hydroxylase pathway was significantly suppressed. Hampered CYPs hydroxylase pathway meant that reduced 20-HETE level, and then the vascular injury from 20-HETE were prevented.

Self-oxidation of Hcy produced O_2_^-^, which could trigger oxidative stress in vascular endothelial, subsequently induced vascular endothelial dysfunction relevant to HHcy. In addition, ROS catalyzed the conversion of LDL to oxidized LDL (ox-LDL), and then accelerated the initiation and progression of atheromatous plaque 8. During vascular endothelial dysfunction, NOX2 oxidase mediated ONOO^-^ formation and oxidized eNOS cofactor tetrahydrobiopterin (BH_4_) to dihydrobiopterin (BH_2_), followed by deficiency of BH_4_. Then eNOS was uncoupled and turned to be a superoxide generating enzyme, and further increased O_2_^-^ production and lowered NO production 4. Moreover, oxidative stress activated RAS and regulated Ang II-induced signal transduction pathways, which play a role in the pathogenesis of vascular endothelial dysfunction.

In current study, according to the western blotting results of NOX2 and eNOS, vascular endothelial oxidative stress was significantly decreased, and ROS induced eNOS uncoupling were remitted. TPE-CA might act as an inhibitor on CYP4A/20-HETE/NOX2 pathway related oxidative stress, result in the reduced eNOS uncoupling and increased NO bioavailability. Furthermore, ACE as a key enzyme for Ang Ⅱ-induced signal transduction pathway was inhibited by TPE-CA. The inhibition of TPE-CA on ACE might exert via lightening the production of ROS and then reducing the activation of ACE by ROS. TPE-CA possessed anti-oxidative stress property that could not only withstand the ROS produced self-oxidation of Hcy, but also suppress the CYPs hydroxylase signal cascade and then reduce the ROS production. In summary, we can reasonably theorize that the regulation property of TPE-CA on CYPs epoxygenase signal cascade and CYPs hydroxylase signal cascade may be a new therapeutic strategy for HHcy-induced vascular endothelial dysfunction.

## Conclusion

**Figure 8:**
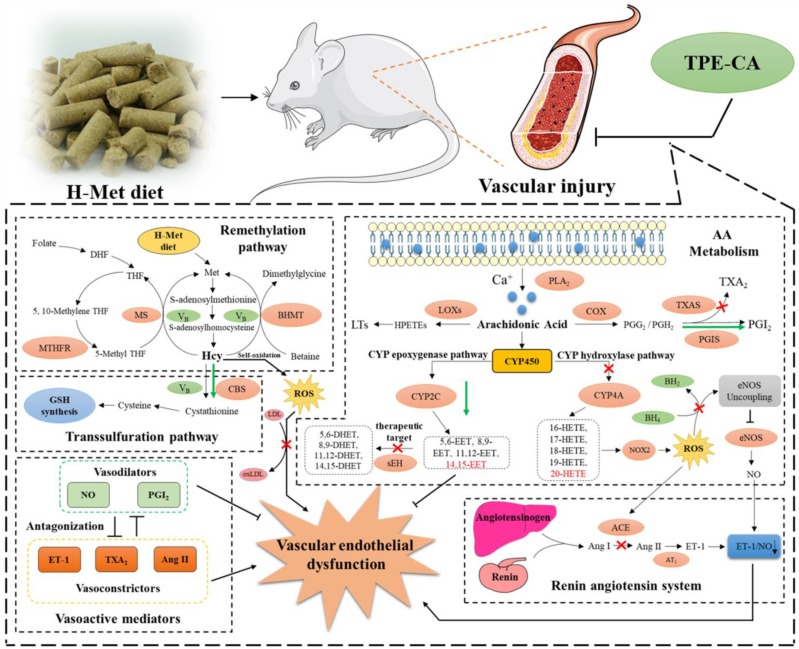
The schematic diagram of therapeutic mechanism of TPE-CA for HHcy-induced vascular endothelial dysfunction. TPE-CA treatment for HHcy could not only effectively reduce the Hcy level in plasma through up-regulating transsulfuration pathway in Hcy metabolism, but also restore the HHcy-induced vascular endothelial dysfunction by activating CYPs epoxygenase signal transduction pathway and inhibiting CYPs hydroxylase signal transduction pathway. Promotion: 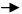 Inhibition: 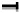 Promotion by TPE-CA: 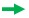
Inhibition by TPE-CA: 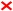

In conclusion, network pharmacology analysis was applied to unravel the underlying therapeutic mechanism of TPE-CA for HHcy-induced vascular endothelial dysfunction. Two pathways of AA metabolism were selected for current experimental validation. As shown in Fig. 8, the treatment of TPE-CA for HHcy could not only effectively reduce the Hcy level in plasma through up-regulating transsulfuration pathway, but also restore the HHcy-induced vascular endothelial dysfunction by activating CYPs epoxygenase signal transduction pathway and inhibiting CYPs hydroxylase signal transduction pathway. Moreover, TPE-CA might suppress oxidative stress in vascular endothelial through CYP4A/20-HETE/NOX2 signaling. These results confirmed that TPE-CA could act on both Hcy metabolism and AA metabolism to ameliorate the HHcy and restore the HHcy-induced vascular endothelial dysfunction. To further elucidate the protection of TPE-CA on HHcy-induced vascular endothelial dysfunction, the detailed molecular mechanisms of anti-oxidative stress property of TPE-CA on HHcy-induced vascular endothelial dysfunction will be deeply investigated in our future studies.

## Supplementary Material

Supplementary figures and tables.

## Abbreviations

Hcy: homocysteine
CVDs: cardiovascular diseases
HHcy: hyperhomocysteinemia
TPE-CA: total phenolic extracts of *Citrus aurantium* L
AA: arachidonic acid
CYPs: cytochrome P450 enzymes
ROS: reactive oxygen species
ACE: angiotensin converting enzyme
LDL: low-density lipoprotein
NO: nitric oxide
ox-LDL: oxidized low-density lipoprotein
eNOS: endothelial nitric oxide synthase
ET-1: endothelin-1
PGI_2_: prostacyclin
PGIS: prostacyclin synthase
TXA_2_: thromboxane A_2_
TXAS: thromboxane A2 synthase
Ang Ⅰ: angiotensin I
Ang Ⅱ: angiotensin Ⅱ
MTHFR: methylenetetrahydrofolate reductase
COX: cyclooxygenase
MS: methionine synthase
LOX: lipoxygenase
BHMT: betaine-homocysteine methyltransferase
EETs: epoxyeicosatrienoic acids
CBS: cystathionine-β-synthase
sEH: soluble epoxide hydrolase
HETEs: hydroxyeicosatetraenoic acids
PVDF: polyvinylidene difluoride
DHETs: dihydroxyeicosatrienoic acids
SDS-PAGE: sodium dodecyl sulfate-polyacrylamide gel electrophoresis
NOX2: NADPH oxidase 2
5,10-MTHF: 5,10-methyltetrahydrofolate
RAS: renin-angiotensin system
BH_4_: tetrahydrobiopterin
BH_2_: dihydrobiopterin
