# Supplementary material for: Vascular Protection of TPE-CA on Hyperhomocysteinemia-induced Vascular Endothelial Dysfunction through AA Metabolism Modulated CYPs Pathway
Source: Int J Biol Sci. 2019 Jul 25;15(10):2037–50. doi: 10.7150/ijbs.35245 (PMC6775291; doi:10.7150/ijbs.35245)
Supplement: Supplementary file 1 — Supplementary figures and tables. [file ijbsv15p2037s1.pdf]

Vascular Protection of TPE-CA on Hyperhomocysteinemia-induced Vascular Endothelial Dysfunction Through AA Metabolism Modulated CYPs Pathway

Hui Li[1], Zhenli Liu[2*], Linlin Liu[1], Wen Li[1], Zhiwen Cao[1], Zhiqian Song[2], Qianqian Yang[1], Aiping Lu[3,4*], Cheng Lu[3*], Yuanyan Liu[1*]

[1] School of Chinese Materia Medica, Beijing University of Chinese Medicine, Beijing 100029, China

[2] Institution of Basic Theory, China Academy of Chinese Medical Sciences, Beijing 100700, China

[3] Institute of Basic Research in Clinical Medicine, China Academy of Chinese Medical Sciences, Beijing 100700, China

[4] School of Chinese Medicine, Hong Kong Baptist University, Kowloon, Hongkong, China

*Correspondence:

Dr. Zhenli Liu: Institution of Basic Theory, China Academy of Chinese Medical Sciences, Beijing 100700, China. Email: zhenli_liu@sina.com.

Dr. Aiping Lu: Institute of Basic Research in Clinical Medicine, China Academy of Chinese Medical Sciences, Beijing 100700, China; School of Chinese Medicine, Hong Kong Baptist University, Kowloon, Hongkong, China. Email: lap64067611@126.com.

Dr. Cheng Lu: Institute of Basic Research in Clinical Medicine, China Academy of Chinese Medical Sciences, Beijing 100700, China. Email: lv_cheng0816@163.com.

Dr. Yuanyan Liu: School of Chinese Materia Medica, Beijing University of Chinese Medicine, Beijing 100029, China. Email: yyliu_1980@163.com.

Contents:

Table S1: The information for MRM parameters.
Table S2: The content (mean ± SD, ng/mL) of 32 prototypical compounds in standard vehicles and plasma sample after oral TPE-CA.
Table S3: The degree of each candidate target.
Table S4: The pathway analysis of 38 screened candidate targets.
Table S5: The interactions of target proteins by STRING database.
Figure S1: MRM chromatograms of each compound (From top to bottom: kaempferitrin, rutin, eriocitrin, diosmetin-7-O-glucoside, narirutin, apigenin-7-O-glucoside, rhoifolin, naringin, diosmin, hesperidin, neohesperidin, xanthotoxol, scoparone, poncirin, eriodictyol, luteolin, apigenin, naringenin, diosmetin, hesperetin, sinensetin, isopimpinellin, limonin, bergapten, isosakuranetin, nomilin, acacetin, nobiletin, tangeretin, 5-demethylnobiletin, auraptene, imperatorin).
Procedure 1: The detailed procedures of enzyme-linked immune sorbent assay.
Procedure 2: The detailed procedures of nitric oxide (NO) determination.

Table S1

No.	Compound	t_R(min)	[M + H]$^+$ (m/z)	MRM Transitions (Precursor → Product)	Fragmentor (V)	Collision Energy (eV)
Time segments: 0~5 min						
01	Kaempferitrin	3.473	579.2	579.2→287.1	100	20
02	Rutin	3.296	611.2	611.2→303.1	100	20
03	Eriocitrin	3.325	597.2	597.2→289	65	15
Time segments: 3.5~8 min						
04	Diosmetin-7-O-Glucoside	3.935	463.1	463.1→153	120	70
05	Narirutin	4.240	581.2	581.2→273.1	95	20
06	Apigenin-7-O-Glucoside	4.396	565.2	565.2→271.1	130	20
07	Rhoifolin	4.403	579.2	579.2→153.1	140	75
08	Naringin	4.743	581.2	581.2→272.9	65	15
09	Diosmin	4.950	609.2	609.2→303	160	35
10	Hesperidin	6.030	611.2	611.2→303.1	90	15
11	Neohesperidin	6.678	611.2	611.2→302.2	90	15
Time segments: 8~15.3 min						
12	Xanthotoxol	9.490	203.1	203.1→129	145	30
13	Scoparone	10.149	207.1	207.1→107.1	125	40
14	Poncirin	12.550	595.2	595.2→287.1	110	20
15	Eriodictyol	12.578	289.1	289.1→153.1	110	25
16	Luteolin	14.418	287.1	287.1→152.9	110	40
Time segments: 15.3~20 min						
17	Apigenin	15.381	271.1	271.1→119.1	130	30
18	Naringenin	14.388	273.1	273.1→153.2	100	20
19	Diosmetin	14.463	301.1	301.1→258.6	140	30
20	Hesperetin	16.549	303.1	303.1→153.1	95	30
21	Sinensetin	16.632	373.2	373.2→343.1	90	25
22	Isopimpinellin	16.569	247.0	247→217	90	35
23	Limonin	16.650	471.2	471.2→425.2	125	15
24	Bergapten	16.742	217.1	217.1→117.9	105	40
25	Isosakuranetin	16.830	287.1	287.1→167.1	115	20
26	Nomilin	17.180	515.3	515.3→161	125	25
27	Acacetin	17.234	285.1	285.1→242.1	130	35
28	Nobiletin	17.266	403.2	403.2→327.1	155	35
29	Tangeretin	17.474	373.2	373.2→343.1	90	25
30	5-Demethylnobiletin	17.642	389.2	389.2→359.1	110	30
31	Auraptene	19.479	299.2	299.2→163	80	10
32	Imperatorin	19.575	271.1	271.1→203	87	15

Table S2

Compounds	Zhishi	
	Extract	Plasma
Acacetin	190.66 ± 12.27	2.06 ± 0.77
Apigenin	359.86 ± 15.6	29.96 ± 6.1
Apigenin-7-O-Glucoside	2858.43±134	12.86 ± 10.92
Auraptene	881.16±54.17	15.15 ± 8.67
Bergapten	670.1±23.4	12.52 ± 2.48
5-Demethylnobiletin	129193.69±6483.34	15.14 ± 7.75
Diosmetin	10032.52±733.33	166.9 ± 27.25
Diosmetin-7-O-Glucoside	8543.58±473.67	608.65 ± 343.65
Diosmin	253815.26±815.34	385.68 ± 70.22
Eriocitrin	295397.93±10570	37.32 ± 13.54
Eriodictyol	1029.4±64	32.08 ± 11.28
Hesperetin	96208.58±3056.66	490.49 ± 187.47
Hesperidin	2757759.6±32313.33	372.14 ± 167.55
Imperatorin	291.87±7.93	3.02 ± 0.71
Isopimpinellin	911.71±60.8	17.06 ± 10.57
Isosakuranetin	306.89±13.48	6.48 ± 1.74
Kaempferitrin	2141.39±42	10.87 ± 5.66
Limonin	32046.15±935.76	47.87 ± 22.96
Luteolin	368136.69±6980	70.63 ± 46.62
Naringenin	111538.49±7303.33	305.68 ± 207.12
Naringin	8881940.01±67363.33	4393.02 ± 2563.65
Narirutin	3515523.8±115793.34	561.58 ± 222.72
Neohesperidin	12324326.22±630973.34	6418.27 ± 2701.27
Nobiletin	743203.27±3033.33	41.67 ± 15.63
Nomilin	13793.84±1051	129.1 ± 14.94
Poncirin	158252.82±1114.33	67.74 ± 7.86
Rhoifolin	313903.34±5026.67	53.88 ± 34.54
Rutin	91943±1470	30.45 ± 18.62
Scoparone	1169.67±37.13	18.67 ± 5.33
Sinensetin	58026.76±3766.67	19.86 ± 8.48
Tangeretin	891688.65±14563.34	55.54 ± 25.49
Xanthotoxol	15768±1068.88	34.7 ± 17.98

Table S3

Name	Degree	Name	Degree
Akt1	21	Fgf2	2
Hmox1	20	Nos2	2
Ppara	17	Ccna2	2
Jun	17	ENSRNOG00000046449	2
Tp53	16	Mme	2
Casp3	14	Prkca	2
Tnf	12	Mitf	2
Cyp1a1	11	Pcna	2
LOC497963	9	Casp1	2
Cyp1a2	9	Casp4	2
Mmp9	8	Slc2a4	1
Fos	8	GLUT3	1
Apob	7	Slc2a3	1
Cyp2c11	7	Slc2a1	1
Cyp2e1	7	Slc2a9	1
Cyp2c22	7	Slc2a7	1
Cyp2c7	7	Akr1c13	1
ENSRNOG00000013241	7	Akr1c12	1
Vegfa	7	Akr1c12l1	1
Jund	7	Akr1c19	1
Smad2	6	Akr1c3	1
Ppard	6	Akr1c1	1
Pparg	6	Tfam	1
Npy2r	6	Nqo1	1
Stat1	6	Gdnf	1
Cyp1b1	5	Dpp4	1
Tlr4	5	Ghsr	1
Cyp19a1	5	Cd80	1
Ptgs2	5	Ghrl	1
Ldlr	5	Irf7	1
Sstr4	5	Ppig	1
Sstr1	5	Nktr	1
Cxcr1	5	Hsdl2	1
Adra2a	5	RGD1564324	1
Il13	5	Decr2	1
Il5	5	Hsd17b14	1
Prkcd	5	Tsta3	1
Prkce	5	LOC682491	1
Tmprss11d	4	Uxs1	1
ENSRNOG00000048887	4	Hsd3b6	1
Bmp2	4	Hsd3b5	1

Esr1	4	Hsd3b	1
Cdk4	4	Hsd3b7	1
Dgat1	4	Nsdhl	1
Notch1	4	Tdh	1
Myc	4	Ugt2b15	1
Prkcg	4	Nkx3-1	1
Sp1	3	Cyp2c24	1
Bcl6	3	Cyp2ac1	1
Bcl6b	3	Soat1	1
Decr1	3	Cck	1
Nfe2l2	3	Ahr	1
Vdr	3	C3	1
Maoa	3	ENSRNOG00000050358	1
Maob	3	Gng2	1
Esr2	3	ENSRNOG00000048272	1
Adipoq	3	Cftr	1
Cdk2	3	Abcc1	1
Sele	3	Mmp8	1
Prkcq	3	Abcb1a	1
Prkcb	3	Kdm1b	1
Ccnd1	3	Kdm1a	1
Slc2a2	2	Abcb4	1
Slc2a5	2		

Table S4

#Term ID	Term description	False discovery rate	Matching proteins in your network (labels)
rno05200	Pathways in cancer	9.54E-15	Akt1, Casp3, Fos, Hmox1, Il13, Il5, Jun, LOC497963, Mmp9, Ppard, Pparg Ptgs2, Smad2, Stat1, Tp53, Vegfa
rno04657	IL-17 signaling pathway	4.86E-12	Casp3, Fos, Il13, Il5, Jun, Jund, Mmp9, Ptgs2, Tnf
rno04933	AGE-RAGE signaling pathway in diabetic complications	6.10E-12	Akt1, Casp3, Jun, Prkcd, Prkce, Smad2, Stat1, Tnf, Vegfa
rno00140	Steroid hormone biosynthesis	1.17E-11	Cyp19a1, Cyp1a1, Cyp1a2, Cyp1b1, Cyp2c11, Cyp2c22, Cyp2c7, Cyp2e1
rno05204	Chemical carcinogenesis	3.09E-11	Cyp1a1, Cyp1a2, Cyp1b1, Cyp2c11, Cyp2c22, Cyp2c7, Cyp2e1, Ptgs2
rno05161	Hepatitis B	3.21E-11	Akt1, Casp3, Fos, Jun, Mmp9, Stat1, Tlr4, Tnf, Tp53
rno05321	Inflammatory bowel disease (IBD)	4.48E-10	Il13, Il5, Jun, Smad2, Stat1, Tlr4, Tnf
rno05140	Leishmaniasis	7.96E-10	Fos, Jun, LOC497963, Ptgs2, Stat1, Tlr4, Tnf
rno05418	Fluid shear stress and atherosclerosis	1.59E-09	Akt1, Fos, Hmox1, Jun, Mmp9, Tnf, Tp53, Vegfa
rno05206	MicroRNAs in cancer	1.79E-09	Casp3, Cyp1b1, Hmox1, Mmp9, Prkce, Ptgs2, Tp53, Vegfa
rno05142	Chagas disease (American trypanosomiasis)	6.85E-09	Akt1, Fos, Jun, LOC497963, Smad2, Tlr4, Tnf
rno04668	TNF signaling pathway	9.26E-09	Akt1, Casp3, Fos, Jun, Mmp9, Ptgs2, Tnf
rno05145	Toxoplasmosis	9.26E-09	Akt1, Casp3, LOC497963, Ldlr, Stat1, Tlr4, Tnf
rno05167	Kaposi's sarcoma-associated herpesvirus infection	1.08E-08	Akt1, Casp3, Fos, Jun, Ptgs2, Stat1, Tp53, Vegfa
rno05205	Proteoglycans in cancer	1.18E-08	Akt1, Casp3, Mmp9, Smad2, Tlr4, Tnf, Tp53, Vegfa
rno04380	Osteoclast differentiation	1.34E-08	Akt1, Fos, Jun, Jund, Pparg, Stat1, Tnf
rno04926	Relaxin signaling pathway	1.77E-08	Akt1, Fos, Jun, LOC497963, Mmp9, Smad2, Vegfa
rno05133	Pertussis	2.35E-08	Casp3, Fos, Jun, LOC497963, Tlr4, Tnf
rno00591	Linoleic acid metabolism	6.46E-08	Cyp1a2, Cyp2c11, Cyp2c22, Cyp2c7, Cyp2e1

rno05210	Colorectal cancer	7.45E-08	Akt1, Casp3, Fos, Jun, Smad2, Tp53
rno04620	Toll-like receptor signaling pathway	8.64E-08	Akt1, Fos, Jun, Stat1, Tlr4, Tnf
rno04010	MAPK signaling pathway	1.98E-07	Akt1, Casp3, Fos, Jun, Jund, Tnf, Tp53, Vegfa
rno04913	Ovarian steroidogenesis	2.67E-07	Cyp19a1, Cyp1a1, Cyp1b1, Ldlr, Ptgs2
rno05160	Hepatitis C	4.19E-07	Akt1, Ldlr, Ppara, Stat1, Tnf, Tp53
rno04210	Apoptosis	4.86E-07	Akt1, Casp3, Fos, Jun, Tnf, Tp53
rno00830	Retinol metabolism	7.36E-07	Cyp1a1, Cyp1a2, Cyp2c11, Cyp2c22, Cyp2c7
rno00590	Arachidonic acid metabolism	1.15E-06	Cyp2c11, Cyp2c22, Cyp2c7, Cyp2e1, Ptgs2
rno04932	Non-alcoholic fatty liver disease (NAFLD)	1.15E-06	Akt1, Casp3, Cyp2e1, Jun, Ppara, Tnf
rno05212	Pancreatic cancer	1.15E-06	Akt1, Smad2, Stat1, Tp53, Vegfa
rno05152	Tuberculosis	1.74E-06	Akt1, Casp3, LOC497963, Stat1, Tlr4, Tnf
rno05323	Rheumatoid arthritis	1.85E-06	Fos, Jun, Tlr4, Tnf, Vegfa
rno04658	Th1 and Th2 cell differentiation	2.50E-06	Fos, Il13, Il5, Jun, Stat1
rno05222	Small cell lung cancer	2.70E-06	Akt1, Casp3, LOC497963, Ptgs2, Tp53
rno01522	Endocrine resistance	2.90E-06	Akt1, Fos, Jun, Mmp9, Tp53
rno05168	Herpes simplex infection	3.86E-06	Casp3, Fos, Jun, Stat1, Tnf, Tp53
rno04066	HIF-1 signaling pathway	3.88E-06	Akt1, Hmox1, LOC497963, Tlr4, Vegfa
rno04660	T cell receptor signaling pathway	3.88E-06	Akt1, Fos, Il5, Jun, Tnf
rno04750	Inflammatory mediator regulation of TRP channels	4.04E-06	Cyp2c11, Cyp2c22, Cyp2c7, Prkcd, Prkce
rno04931	Insulin resistance	4.25E-06	Akt1, Ppara, Prkcd, Prkce, Tnf
rno05165	Human papillomavirus infection	4.25E-06	Akt1, Casp3, Ptgs2, Stat1, Tnf, Tp53, Vegfa
rno04726	Serotonergic synapse	6.92E-06	Casp3, Cyp2c11, Cyp2c22, Cyp2c7, Ptgs2
rno04915	Estrogen signaling pathway	8.94E-06	Akt1, Fos, Jun, Mmp9, Prkcd
rno05162	Measles	9.43E-06	Akt1, Il13, Stat1, Tlr4, Tp53

rno00980	Metabolism of xenobiotics by cytochrome P450	1.45E-05	Cyp1a1, Cyp1a2, Cyp1b1, Cyp2e1
rno05166	HTLV-I infection	1.95E-05	Akt1, Fos, Jun, Smad2, Tnf, Tp53
rno04664	Fc epsilon RI signaling pathway	2.11E-05	Akt1, Il13, Il5, Tnf
rno04621	NOD-like receptor signaling pathway	2.12E-05	Jun, Prkcd, Stat1, Tlr4, Tnf
rno05164	Influenza A	2.21E-05	Akt1, Jun, Stat1, Tlr4, Tnf
rno05132	Salmonella infection	3.92E-05	Fos, Jun, LOC497963, Tlr4
rno04024	cAMP signaling pathway	5.78E-05	Akt1, Fos, Jun, Ppara, Sstr1
rno05310	Asthma	6.38E-05	Il13, Il5, Tnf
rno05146	Amoebiasis	6.84E-05	Casp3, LOC497963, Tlr4, Tnf
rno01100	Metabolic pathways	9.01E-05	Cyp19a1, Cyp1a1, Cyp1a2, Cyp2c11, Cyp2c22, Cyp2c7, Cyp2e1 Hmox1, LOC497963, Ptgs2
rno04659	Th17 cell differentiation	9.01E-05	Fos, Jun, Smad2, Stat1
rno04060	Cytokine-cytokine receptor interaction	0.0001	Cxcr1, Il13, Il5, Tnf, Vegfa
rno04071	Sphingolipid signaling pathway	0.00015	Akt1, Prkce, Tnf, Tp53
rno04722	Neurotrophin signaling pathway	0.00015	Akt1, Jun, Prkcd, Tp53
rno05219	Bladder cancer	0.00015	Mmp9, Tp53, Vegfa
rno00380	Tryptophan metabolism	0.00018	Cyp1a1, Cyp1a2, Cyp1b1
rno04371	Apelin signaling pathway	0.0002	Akt1, LOC497963, Prkce, Smad2
rno04930	Type II diabetes mellitus	0.00021	Prkcd, Prkce, Tnf
rno05014	Amyotrophic lateral sclerosis (ALS)	0.00027	Casp3, Tnf, Tp53
rno05134	Legionellosis	0.00028	Casp3, Tlr4, Tnf
rno05224	Breast cancer	0.00029	Akt1, Fos, Jun, Tp53
rno04630	Jak-STAT signaling pathway	0.0003	Akt1, Il13, Il5, Stat1
rno04370	VEGF signaling pathway	0.00035	Akt1, Ptgs2, Vegfa

rno05225	Hepatocellular carcinoma	0.00046	Akt1, Hmox1, Smad2, Tp53
rno04062	Chemokine signaling pathway	0.00049	Akt1, Cxcr1, Prkcd, Stat1
rno05211	Renal cell carcinoma	0.00055	Akt1, Jun, Vegfa
rno04662	B cell receptor signaling pathway	0.00056	Akt1, Fos, Jun
rno04920	Adipocytokine signaling pathway	0.00056	Akt1, Ppara, Tnf
rno04917	Prolactin signaling pathway	0.00062	Akt1, Fos, Stat1
rno01524	Platinum drug resistance	0.00071	Akt1, Casp3, Tp53
rno03320	PPAR signaling pathway	0.00073	Ppara, Ppard, Pparg
rno04666	Fc gamma R-mediated phagocytosis	0.00096	Akt1, Prkcd, Prkce
rno04211	Longevity regulating pathway	0.001	Akt1, Pparg, Tp53
rno04064	NF-kappa B signaling pathway	0.0011	Ptgs2, Tlr4, Tnf
rno05215	Prostate cancer	0.0013	Akt1, Mmp9, Tp53
rno05231	Choline metabolism in cancer	0.0014	Akt1, Fos, Jun
rno04919	Thyroid hormone signaling pathway	0.0018	Akt1, Stat1, Tp53
rno04080	Neuroactive ligand-receptor interaction	0.0026	Adra2a, Npy2r, Sstr1, Sstr4
rno04217	Necroptosis	0.0035	Stat1, Tlr4, Tnf
rno04310	Wnt signaling pathway	0.0035	Jun, Ppard, Tp53
rno05216	Thyroid cancer	0.0036	Pparg, Tp53
rno04921	Oxytocin signaling pathway	0.0037	Fos, Jun, Ptgs2
rno05226	Gastric cancer	0.0039	Akt1, Smad2, Tp53
rno04216	Ferroptosis	0.0041	Hmox1, Tp53
rno04151	PI3K-Akt signaling pathway	0.0043	Akt1, Tlr4, Tp53, Vegfa
rno04022	cGMP-PKG signaling pathway	0.0047	Adra2a, Akt1, Prkce
rno05202	Transcriptional misregulation in cancer	0.0052	Mmp9, Pparg, Tp53

rno04218	Cellular senescence	0.0056	Akt1, Smad2, Tp53
rno04979	Cholesterol metabolism	0.006	Apob, Ldlr
rno05016	Huntington's disease	0.0066	Casp3, Pparg, Tp53
rno05144	Malaria	0.0066	Tlr4, Tnf
rno04923	Regulation of lipolysis in adipocytes	0.007	Akt1, Ptgs2
rno04510	Focal adhesion	0.0073	Akt1, Jun, Vegfa
rno05330	Allograft rejection	0.0074	Il5, Tnf
rno05203	Viral carcinogenesis	0.0078	Casp3, Jun, Tp53
rno05213	Endometrial cancer	0.0078	Akt1, Tp53
rno00982	Drug metabolism - cytochrome P450	0.0081	Cyp1a2, Cyp2e1
rno05230	Central carbon metabolism in cancer	0.0081	Akt1, Tp53
rno04137	Mitophagy - animal	0.0082	Jun, Tp53
rno05031	Amphetamine addiction	0.0084	Fos, Jun
rno05169	Epstein-Barr virus infection	0.0085	Akt1, Jun, Tp53
rno04115	p53 signaling pathway	0.009	Casp3, Tp53
rno05221	Acute myeloid leukemia	0.0092	Akt1, Ppard
rno05223	Non-small cell lung cancer	0.0092	Akt1, Tp53
rno05214	Glioma	0.0093	Akt1, Tp53
rno05218	Melanoma	0.0106	Akt1, Tp53
rno05220	Chronic myeloid leukemia	0.0122	Akt1, Tp53
rno01521	EGFR tyrosine kinase inhibitor resistance	0.0124	Akt1, Vegfa
rno04012	ErbB signaling pathway	0.0137	Akt1, Jun
rno04144	Endocytosis	0.0137	Cxcr1, Ldlr, Smad2
rno04350	TGF-beta signaling pathway	0.0137	Smad2, Tnf
rno04912	GnRH signaling pathway	0.0138	Jun, Prkcd

rno04640	Hematopoietic cell lineage	0.0143	Il5, Tnf
rno04650	Natural killer cell mediated cytotoxicity	0.0148	Casp3, Tnf
rno04922	Glucagon signaling pathway	0.0148	Akt1, Ppara
rno04925	Aldosterone synthesis and secretion	0.0148	Ldlr, Prkce
rno04725	Cholinergic synapse	0.02	Akt1, Fos
rno04270	Vascular smooth muscle contraction	0.0235	Prkcd, Prkce
rno04152	AMPK signaling pathway	0.0253	Akt1, Pparg
rno04728	Dopaminergic synapse	0.0258	Akt1, Fos
rno04110	Cell cycle	0.026	Smad2, Tp53
rno04068	FoxO signaling pathway	0.0266	Akt1, Smad2
rno04140	Autophagy - animal	0.0268	Akt1, Prkcd
rno04550	Signaling pathways regulating pluripotency of stem cells	0.0289	Akt1, Smad2
rno04072	Phospholipase D signaling pathway	0.0341	Akt1, Cxcr1
rno04150	mTOR signaling pathway	0.0351	Akt1, Tnf
rno04530	Tight junction	0.0375	Jun, Prkce
rno05010	Alzheimer's disease	0.0417	Casp3, Tnf

Table S5

#Node1	Node2	Homology	Coexpression	Experimentally_determined_ interaction	Database_annotated	Automated_textmining	Combined_score
Jun	Fos	0	0.761	0.86	0.9	0.953	0.999
Apob	Ldlr	0	0.063	0.306	0.9	0.932	0.995
Jund	Fos	0	0.064	0.765	0.8	0.876	0.993
Tp53	Akt1	0	0.049	0.07	0.9	0.922	0.992
Vegfa	Mmp9	0	0	0.068	0.8	0.925	0.985
Cyp2e1	Ptgs2	0	0.063	0.046	0.9	0.682	0.967
Stat1	Il13	0	0	0	0.9	0.64	0.962
Cyp2c22	Cyp1a2	0.764	0.495	0	0.9	0.322	0.955
Jun	Tnf	0	0.049	0.079	0.8	0.757	0.951
Tp53	Casp3	0	0.063	0.078	0	0.945	0.948
Akt1	Casp3	0	0.05	0.203	0	0.936	0.948
Cyp2c7	Cyp1a2	0.764	0.339	0	0.9	0.588	0.946
Cyp1a1	Cyp19a1	0.569	0	0	0.9	0.66	0.939
Cyp1b1	Cyp1a1	0.842	0.321	0	0.9	0.932	0.939
Apob	Tlr4	0	0	0.147	0.9	0.345	0.939
Cyp1a2	Cyp2e1	0.767	0.176	0	0.9	0.898	0.938
Akt1	Vegfa	0	0.063	0.164	0	0.923	0.935
Prkcd	Casp3	0	0	0.144	0.9	0.298	0.934
Cyp1a2	Cyp2c11	0.761	0.101	0	0.9	0.87	0.933
Cyp1a1	Cyp2e1	0.793	0.092	0	0.9	0.881	0.928
Prkcd	Stat1	0	0.063	0.181	0.9	0.146	0.925
Sstr1	Npy2r	0.7	0.129	0	0.9	0.429	0.92

Cyp2c22	Cyp2e1	0.948	0.174	0	0.9	0.602	0.918
Adra2a	Sstr4	0.63	0.063	0	0.9	0.467	0.918
Cyp2c7	Cyp2e1	0.955	0.168	0	0.9	0.623	0.917
Il5	Il13	0	0.063	0	0	0.914	0.916
Sstr1	Adra2a	0.622	0.08	0	0.9	0.347	0.915
Sstr1	Sstr4	0.957	0.154	0	0.9	0.879	0.915
Tlr4	Tnf	0	0.063	0.044	0	0.911	0.913
Npy2r	Sstr4	0.715	0.117	0	0.9	0.205	0.912
Cyp2c11	Cyp2e1	0.958	0.064	0	0.9	0.874	0.907
Prkcd	Prkce	0.908	0	0	0.9	0.818	0.907
Npy2r	Adra2a	0.59	0.063	0	0.9	0.15	0.906
Sstr4	Cxcr1	0.766	0.079	0	0.9	0	0.903
Sstr1	Cxcr1	0.764	0	0	0.9	0.084	0.901
Npy2r	Cxcr1	0.619	0	0	0.9	0.081	0.901
Adra2a	Cxcr1	0.579	0	0	0.9	0	0.9
Casp3	Tnf	0	0	0.547	0	0.775	0.893
Ptgs2	Tnf	0	0.064	0	0	0.888	0.891
Tp53	Ptgs2	0	0	0.163	0	0.87	0.887
Cyp1b1	Cyp1a2	0.815	0.321	0	0.8	0.863	0.88
Akt1	Hmox1	0	0	0.109	0	0.869	0.879
Smad2	Akt1	0	0.063	0.488	0	0.769	0.879
Akt1	Mmp9	0	0	0	0	0.877	0.877
Mmp9	Tnf	0	0.117	0.071	0	0.858	0.873
Il13	Tnf	0	0.063	0	0	0.859	0.862
Akt1	LOC497963	0	0	0.191	0.6	0.599	0.859
Akt1	Jun	0	0	0.094	0	0.846	0.854

Hmox1	Ptgs2	0	0.063	0	0	0.85	0.854
Vegfa	Tnf	0	0	0	0	0.852	0.852
Akt1	Tnf	0	0	0.11	0	0.834	0.846
Vegfa	Ptgs2	0	0.064	0	0	0.841	0.845
Akt1	Ptgs2	0	0	0.051	0	0.834	0.837
LOC497963	Ptgs2	0	0.063	0.255	0	0.775	0.829
Tp53	Vegfa	0	0	0	0	0.827	0.827
Tp53	Hmox1	0	0.066	0	0	0.82	0.825
Mmp9	Tlr4	0	0.101	0	0	0.81	0.823
Jund	Jun	0.924	0.064	0.058	0.8	0.868	0.82
Tp53	Jun	0	0	0.054	0	0.817	0.82
Cyp1a1	Cyp1a2	0.972	0.063	0	0.8	0.893	0.811
Fos	Ptgs2	0	0.064	0	0	0.806	0.811
Il5	Tnf	0	0	0	0	0.804	0.804
Hmox1	Tlr4	0	0.058	0	0	0.794	0.798
Mmp9	Jun	0	0	0.152	0	0.769	0.796
Hmox1	Casp3	0	0	0	0	0.792	0.792
Stat1	Jun	0	0	0.219	0	0.734	0.783
Tp53	Tnf	0	0	0	0	0.78	0.78
Tlr4	Ptgs2	0	0.101	0	0	0.761	0.776
Akt1	Fos	0	0	0.085	0	0.762	0.773
LOC497963	Tnf	0	0.064	0	0	0.766	0.772
Mmp9	Il13	0	0	0	0	0.765	0.765
Tp53	Mmp9	0	0	0	0	0.763	0.763
Akt1	Stat1	0	0	0.155	0	0.726	0.759
Stat1	Fos	0	0	0.305	0	0.664	0.756

Tp53	Cyp1a1	0	0	0	0	0.752	0.752
Il13	Ptgs2	0	0	0	0	0.752	0.752
Vegfa	Il13	0	0	0	0	0.751	0.751
LOC497963	Tlr4	0	0.159	0	0	0.711	0.746
Mmp9	Ptgs2	0	0.064	0	0	0.739	0.745
Casp3	Jun	0	0	0.124	0	0.72	0.744
Cyp1a1	Cyp2c11	0.787	0.063	0	0.65	0.86	0.744
Tp53	Fos	0	0	0.083	0	0.726	0.738
Akt1	Tlr4	0	0	0.127	0	0.711	0.736
Casp3	Tlr4	0	0	0.158	0	0.698	0.734
Mmp9	Casp3	0	0.064	0	0	0.724	0.73
Pparg	Ptgs2	0	0	0	0	0.729	0.729
Tp53	Stat1	0	0	0.089	0	0.714	0.728
Tlr4	Jun	0	0	0	0	0.725	0.725
Cyp2c7	Cyp1a1	0.795	0.063	0	0.65	0.596	0.724
Smad2	Mmp9	0	0	0.216	0	0.66	0.722
Vegfa	Jun	0	0.063	0.217	0	0.651	0.721
Cyp2c22	Cyp2c7	0.966	0.207	0	0.65	0.732	0.721
Cyp2c22	Cyp1a1	0.781	0.063	0	0.65	0.448	0.718
Casp3	Ptgs2	0	0	0	0	0.717	0.717
Vegfa	Casp3	0	0	0	0	0.714	0.714
Fos	Tnf	0	0.044	0	0	0.711	0.712
Pparg	Tnf	0	0	0	0	0.709	0.709
Cyp2c11	Ptgs2	0	0.063	0.046	0.65	0.179	0.708
Akt1	Pparg	0	0	0.136	0	0.676	0.708
Vegfa	Tlr4	0	0	0	0	0.701	0.701

Smad2	Tp53	0	0	0.219	0	0.634	0.701
Tlr4	Il13	0	0	0	0	0.695	0.695
Cyp2e1	Jun	0	0	0.446	0	0.471	0.694
Mmp9	Hmox1	0	0.063	0	0	0.686	0.694
Cyp1b1	Cyp19a1	0	0	0	0	0.693	0.693
Stat1	Tlr4	0	0.063	0.109	0	0.658	0.689
Stat1	Casp3	0	0.063	0.15	0	0.639	0.687
Cyp2c22	Ptgs2	0	0.063	0.046	0.65	0.101	0.681
Hmox1	Tnf	0	0.063	0	0	0.671	0.679
Tp53	Pparg	0	0	0.069	0	0.667	0.677
Hmox1	Cyp2e1	0	0.139	0	0	0.635	0.672
Cyp2c7	Ptgs2	0	0.063	0.046	0.65	0.077	0.672
Stat1	Tnf	0	0.063	0	0	0.662	0.67
Vegfa	Stat1	0	0	0.158	0	0.618	0.665
Hmox1	LOC497963	0	0	0.043	0	0.663	0.663
Cyp2c22	Cyp2c11	0.964	0	0	0.65	0.514	0.661
Cyp2c7	Cyp2c11	0.975	0	0	0.65	0.792	0.66
Stat1	Ptgs2	0	0	0	0	0.656	0.656
Jun	Ptgs2	0	0.063	0	0	0.642	0.65
Smad2	Jun	0	0	0.283	0	0.528	0.647
LOC497963	Casp3	0	0	0	0	0.644	0.644
Tlr4	Il5	0	0	0	0	0.643	0.643
Akt1	Cyp19a1	0	0	0.048	0	0.638	0.64
Vegfa	Pparg	0	0	0	0	0.635	0.635
Vegfa	Hmox1	0	0	0	0	0.626	0.626
Casp3	Fos	0	0	0	0	0.626	0.626

Tlr4	Pparg	0	0.104	0.061	0	0.591	0.625
Vegfa	Il5	0	0	0	0	0.624	0.624
Mmp9	LOC497963	0	0.169	0	0	0.562	0.62
Mmp9	Fos	0	0.063	0.054	0	0.601	0.616
Mmp9	Stat1	0	0	0.057	0	0.609	0.615
LOC497963	Il13	0	0	0	0	0.615	0.615
Ptgs2	Cyp19a1	0	0.049	0.046	0	0.61	0.615
Stat1	LOC497963	0	0.05	0	0	0.611	0.614
Smad2	Casp3	0	0.063	0	0	0.597	0.606
Cyp1a1	Hmox1	0	0.139	0	0	0.539	0.586
Stat1	Il5	0	0	0	0	0.585	0.585
Apob	Ppara	0	0.099	0.051	0	0.551	0.582
LOC497963	Jun	0	0	0	0	0.582	0.582
Mmp9	Pparg	0	0	0	0	0.571	0.571
Vegfa	LOC497963	0	0.064	0	0	0.555	0.565
Tlr4	Fos	0	0.113	0.05	0	0.522	0.562
Pparg	Fos	0	0	0.078	0	0.543	0.561
Tp53	Tlr4	0	0.063	0.05	0	0.546	0.561
Akt1	Ldlr	0	0	0.069	0	0.548	0.561
Stat1	Pparg	0	0.064	0.152	0	0.488	0.558
Smad2	Fos	0	0	0.154	0	0.494	0.554
Tp53	Cyp19a1	0	0	0	0	0.552	0.552
Ppara	Cyp2e1	0	0.13	0.087	0	0.479	0.551
Cyp1b1	Ptgs2	0	0.063	0.046	0	0.537	0.55
Ppara	Akt1	0	0	0.136	0	0.498	0.547
Cyp2e1	Casp3	0	0	0	0	0.547	0.547

Hmox1	Jun	0	0	0	0	0.543	0.543
Casp3	Pparg	0	0	0.076	0	0.524	0.541
Akt1	Il13	0	0	0	0	0.54	0.54
Apob	Akt1	0	0	0.111	0	0.499	0.535
Ppara	Ptgs2	0	0	0	0	0.535	0.535
Fos	Cyp19a1	0	0	0	0	0.532	0.532
Akt1	Il5	0	0	0	0	0.531	0.531
Ppara	Ldlr	0	0	0	0	0.528	0.528
Smad2	Vegfa	0	0	0	0	0.524	0.524
Tnf	Cyp19a1	0	0	0	0	0.517	0.517
Cyp1a1	Ptgs2	0	0.063	0.046	0	0.495	0.509
Cyp2e1	Tnf	0	0	0	0	0.509	0.509
Cyp1b1	Tp53	0	0	0	0	0.507	0.507
Stat1	Hmox1	0	0	0	0	0.507	0.507
Ldlr	Pparg	0	0	0	0	0.504	0.504
Hmox1	Fos	0	0	0	0	0.504	0.504
Tp53	Cyp2e1	0	0	0	0	0.5	0.499
Casp3	Cyp19a1	0	0	0	0	0.498	0.497
Cxcr1	Tnf	0	0.063	0	0	0.481	0.493
Vegfa	Fos	0	0	0	0	0.492	0.492
Mmp9	Il5	0	0	0	0	0.492	0.491
Smad2	Stat1	0	0.063	0.069	0	0.455	0.483
Tp53	Cyp1a2	0	0	0	0	0.481	0.481
Hmox1	Pparg	0	0	0	0	0.481	0.481
Apob	Pparg	0	0.063	0.051	0	0.46	0.478
Cyp2e1	Tlr4	0	0	0.042	0	0.478	0.478

Casp3	Il13	0	0	0	0	0.472	0.472
Cyp1b1	Hmox1	0	0.139	0	0	0.404	0.464
Cxcr1	Akt1	0	0	0.057	0	0.453	0.462
Cyp1a2	Hmox1	0	0.139	0	0	0.398	0.459
Cxcr1	Mmp9	0	0.064	0.055	0	0.436	0.458
Jund	Cyp2e1	0	0	0.446	0	0.055	0.454
Il5	Ptgs2	0	0	0	0	0.453	0.453
Smad2	Pparg	0	0	0.159	0	0.354	0.434
Smad2	Tlr4	0	0.081	0.07	0	0.39	0.433
Ppara	Tnf	0	0	0	0	0.433	0.433
Tlr4	Ldlr	0	0	0.057	0	0.418	0.428
Pparg	Jun	0	0	0.054	0	0.42	0.428
Akt1	Cyp2e1	0	0	0.048	0	0.419	0.423
Tp53	Prkcd	0	0	0.163	0	0.337	0.421
Ppard	Akt1	0	0.063	0.136	0	0.343	0.421
Mmp9	Ldlr	0	0	0.05	0	0.413	0.419
Hmox1	Il13	0	0	0	0	0.419	0.418
Tp53	LOC497963	0	0	0	0	0.417	0.417
Fos	Il13	0	0	0	0	0.415	0.415
Ldlr	Jun	0	0	0	0	0.412	0.412
Cxcr1	Tlr4	0	0.064	0.044	0	0.394	0.411
LOC497963	Pparg	0	0.099	0.142	0	0.294	0.407
Ppara	Cyp1a2	0	0.088	0.087	0	0.345	0.407
Pparg	Cyp19a1	0	0	0.087	0	0.377	0.407
Vegfa	Cyp19a1	0	0	0	0	0.405	0.405
Smad2	Tnf	0	0	0	0	0.405	0.405

Ldlr	Tnf	0	0.05	0	0	0.399	0.404
Apob	Cyp2e1	0	0.218	0	0	0.265	0.4

Figure S1

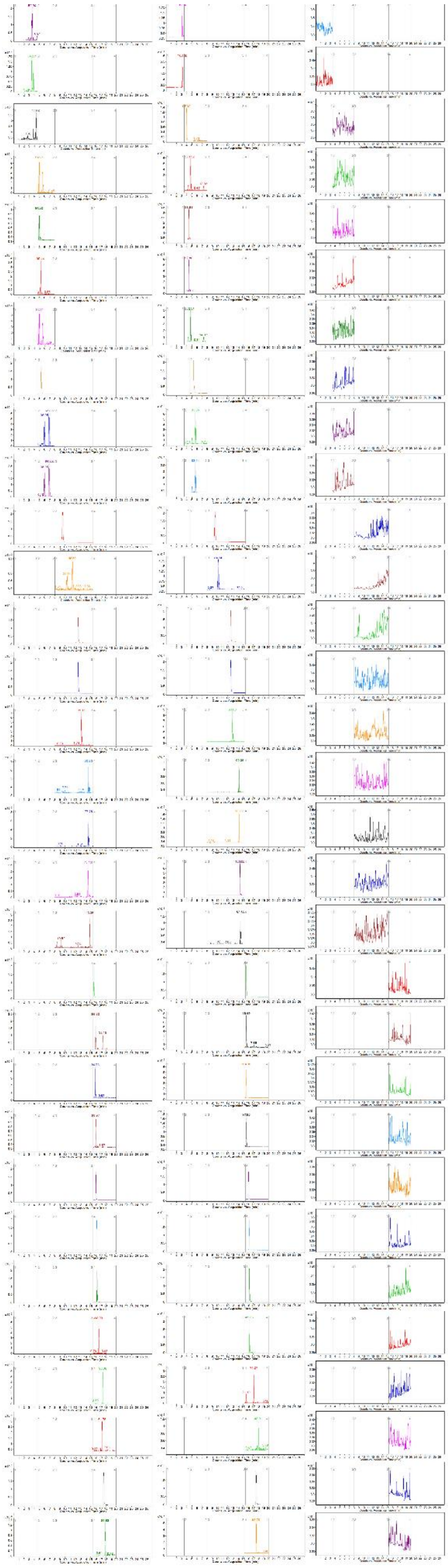

Procedure 1

The detailed procedures of enzyme-linked immune sorbent assay

The kit was stabilized for half hour in ambient temperature, then 50 μL diluted standard and sample were added into standard well and sample well, respectively. Biotinylated Ab working solution was added into both standard well and sample well, then sealed with closure plate membrane and shook gently followed by incubating for 30 minutes at 37 °C. After the incubation, the membrane was removed carefully and the liquid in the wells were drained. Then, the washing solution was added into each well, and discarded after stabilizing 30 seconds. This process was repeated 5 times, then patted to dry. Following washing, 50 μL HRP working solution was added into each standard well and sample well, then shook gently after sealed with closure plate membrane, and then incubated for 30 minutes at 37 °C. After the incubation, washing process was carried 5 times as abovementioned. Afterwards, 50 μL chromogenic solution A and 50 μL chromogenic solution B were successively added into each well. After gently shook to blended, it was incubated for 10 minutes at 37 °C in dark environment. After the incubation, 50 μL stop solution was added into each well to stop the reaction. Then, the optical density (OD) of each well under 450 nm wavelength was measured within 10 minutes. In empty well, it only adds chromogen solution A, B and stop solution for zero setting. The experiment was performed in triplicate. Each sample was set three wells. And the mean OD of the three wells was used in the data calculation.

Procedure 2

The detailed procedures of nitric oxide (NO) determination

In general, 0.1 mL double distilled water, 0.1 mL standard solution (100 μmol/L) and 0.1 mL sample were added into blank test tube, standard test tube and sample test tube, respectively. Then, 0.4 mL mixed reagent contained in assay kit was added into test tube followed by incubating at 37 °C for 1 h. After incubation, 0.2 mL stop solution and 0.1 mL clarifying solution were successively added into these test tubes, vortexed for 30 s, and then stabilized for 40 minutes at room temperature. Afterwards, all test tubes were centrifuged at 4000 rpm for 10 min. Equal volumes of the supernatant (0.5 mL) was fetched, and then 0.6 mL chromogenic reagent was added into test tubes. After stabilized for 10 minutes at room temperature, the OD value of each tube under 550 nm wavelength was determined. The experiment was performed in triplicate, each sample set three test tubes, and the mean OD of the three test tubes was used in the data calculation. Calculation formula showed in Equation 1. Blank test tubes were used for zero setting. All procedures were performed according to the manufacturer's specifications.

$$C_{(NO)} = \frac{OD_{(S)} - OD_{(B)}}{OD_{(St)} - OD_{(B)}} \times C_{(St)} \times N \qquad \text{Equation 1}$$

Where $C_{(NO)}$ is the concentration of NO, the unit is *μmol/L*, $OD_{(S)}$ is the OD value of samples, $OD_{(B)}$ is the OD value of double distilled water, $OD_{(St)}$ is the OD value of standards, $C_{(St)}$ is the concentration of standards = 100 μmol/L, *N* is the dilution multiple of samples (in present experiment, *N*=10).

Detailed information for target compounds determination

The detailed information as follows: A Poroshell 120 SB-C18 column (4.6 mm × 100 mm; 2.7 μm) was used for the chromatographic separation, the injected volume of sample was 2 μL and the column temperature was maintained at 25 °C. TPE-CA samples were eluted with 5 % acetonitrile (v/v in water) and 0.1 % (v/v) formic acid, the flow rate was 0.5 mL/min. The positive ion modes were performed with MRM for quantitative analysis by ESI. The mass spectrometer was operated in positive mode, with the primary parameters being set as follows: capillary voltage was set at 4000V, corona current was maintained at 10 nA, drying gas temperature was set at 300 °C and the sheath gas temperature was maintained at 250 °C. Nebulizer pressure was set at 45 psi, drying gas

flow rate was maintained at 11 L/min and sheath gas flow rate was maintained at 7 L/min.
